# Augmented Movelet Method for Activity Classification Using Smartphone Gyroscope and Accelerometer Data

**DOI:** 10.3390/s20133706

**Published:** 2020-07-02

**Authors:** Emily J. Huang, Jukka-Pekka Onnela

**Affiliations:** 1 Department of Mathematics and Statistics, Wake Forest University, Winston Salem, NC 27106, USA; 2 Department of Biostatistics, Harvard T.H. Chan School of Public Health, Harvard University, Boston, MA 02115, USA; onnela@hsph.harvard.edu

**Keywords:** activity level, activity recognition, digital phenotyping

## Abstract

Physical activity, such as walking and ascending stairs, is commonly used in biomedical settings as an outcome or covariate. Researchers have traditionally relied on surveys to quantify activity levels of subjects in both research and clinical settings, but surveys are subjective in nature and have known limitations, such as recall bias. Smartphones provide an opportunity for unobtrusive objective measurement of physical activity in naturalistic settings, but their data tends to be noisy and needs to be analyzed with care. We explored the potential of smartphone accelerometer and gyroscope data to distinguish between walking, sitting, standing, ascending stairs, and descending stairs. We conducted a study in which four participants followed a study protocol and performed a sequence of activities with one phone in their front pocket and another phone in their back pocket. The subjects were filmed throughout, and the obtained footage was annotated to establish moment-by-moment ground truth activity. We introduce a modified version of the so-called movelet method to classify activity type and to quantify the uncertainty present in that classification. Our results demonstrate the promise of smartphones for activity recognition in naturalistic settings, but they also highlight challenges in this field of research.

## 1. Introduction

Many researchers have recently advocated for a more substantial role for large-scale phenotyping as a route to advances in the biomedical sciences. Of the many different phenotype classes, precise capture of social, behavioral, and cognitive markers in naturalistic settings has traditionally presented special challenges to phenomics because of their temporal nature, contextual dependence, and lack of tools for measuring them objectively. The ubiquity of smartphones presents an opportunity to capture these markers in free-living settings, offering a scalable solution to the phenotyping problem [1,2]. Smartphones have at least three distinct advantages compared to other approaches to social, behavioral, and cognitive phenotyping: (1) the availability of these devices makes it possible to implement large studies without requiring additional subject instrumentation; (2) reliance on sensor data makes the process unobtrusive and poses no burden on the subject, making long-term followup possible; and (3) the combination of the previous two factors makes it possible, at least in principle, to obtain these markers prospectively from a cohort of interest at a low cost.

The creation of more precise and nuanced behavioral digital phenotypes is not only important but also very timely [3]. In particular, physical activity level monitoring has applications in a variety of medical areas [4]. For example, in psychiatry, activity monitoring can be used to assess and to detect changes in depression severity [5]. For oncology patients, mobility monitoring after surgery can be used to quantify the expected postoperative recovery course and to identify patients who deviate from this course and may be experiencing postoperative complications [6,7]. Activity level monitoring can also be used to assess the effectiveness of interventions designed to increase physical activity levels [8]. Traditionally, physical activity levels have been monitored using subject self-report through questionnaires, surveys, interviews, and diaries. However, these methods can underestimate or overestimate true activity levels since they are subject to recall and response bias [8,9,10,11,12]. Self-report methods can also be burdensome to the subjects who must complete them, which may reduce compliance [10,13].

To classify different types of physical activity, we consider data from the tri-axial accelerometer and tri-axial gyroscope in a smartphone. These sensors output data from three orthogonal axes. In our dataset, the axes are in the frame of reference of the phone, with the *x*-axis running from left to right across the screen of the phone, the *y*-axis from bottom to top across the screen, and the *z*-axis perpendicular to the screen. The gyroscope measures the angular velocity about each axis, which indicates the direction and speed that the phone is spinning about the axis. The accelerometer measures the acceleration along each axis. Throughout this paper, angular velocity is given in units of radians per second (radians/second) and acceleration in units of *g* (1 *g* = 9.81 m/s/s). In the study dataset that is analyzed in this paper, we collected iPhone accelerometer and gyroscope data at 10 Hz, i.e., 10 samples per second.

The starting point of our investigation is the so-called movelet method, which was developed by Bai et al. [14] for activity recognition using wearable device data. The method was designed to infer the activity that a person is performing based on data from a tri-axial accelerometer worn at the hip. A small amount of training data (i.e., data with ground-truth activity labels) is collected on a subject and used to construct a dictionary. The dictionary can then be used to make activity classifications for the subject’s incoming unlabeled data. The procedure is described in detail in Section 2. Since the development of the movelet method, further extensions have been proposed. He et al. [15] extended the movelet method to incorporate three accelerometers worn at the right hip, left wrist, and right wrist, respectively. Xiao et al. [16] extended the movelet method to perform activity recognition for a given subject using another person’s dictionary. In this paper, we propose a new extension to the method by Bai et al. [14], allowing estimation of the accuracy of the outputted activity classifications.

There has been a growing literature on using smartphone accelerometer or gyroscope data to perform activity recognition (see Straczkiewicz and Onnela [17] and Trifan et al. [18] for systematic reviews on this topic). A common approach is to first extract features from the data (such as the mean and standard deviation of the acceleration data along a certain axis) and then use those features as covariates in a classifier. A variety of classifiers have been tested, such as logistic regression, decision trees, k-nearest neighbors, support vector machine, random forest, and neural networks [17,19,20,21,22]. We investigated the movelet method in this paper because it is designed to distinguish between different activities using continuous data from easily accessible sensors. The method is transparent and interpretable. The dictionaries are personalized to each subject, incorporating their unique activity data. Moreover, the method requires less training data compared to the aforementioned methods [14]. In our analysis, we built each subject’s dictionary using only a few seconds of their training data from each activity.

Few studies have investigated the performance of the movelet method in smartphone data. Previous studies on the movelet method largely focused on accelerometry data from wearable devices [14,15,16]. To the best of our knowledge, only the study by Martin et al. [23] has tested the movelet method on smartphone data. In their work, they applied the method to distinguish between various transportation modes (e.g., traveling by bus, car, train, or foot) using smartphone accelerometer and GPS data. In this paper, we consider smartphone accelerometer and gyroscope data, and we focus on other activities that are common to daily life, including walking at different speeds, sitting, standing, ascending stairs, and descending stairs.

The main contributions of our paper include (i) the application of the movelet method to smartphone data, (ii) the application of the movelet method to gyroscope data while previous analyses have been limited to accelerometer and GPS data, and (iii) an extension of the movelet method to quantify uncertainty associated with the activity classifications. In (i) and (ii), we implemented the original movelet method proposed by Bai et al. [14], as well as variations of this method using alternative distance metrics and data transformations. The data used in this paper were collected from an experiment that we conducted where four healthy participants performed various activities while wearing two smartphones and three wearable devices. The present paper focuses on accelerometer and gyroscope data from the two smartphones; joint analyses of the smartphone and wearable data will be discussed elsewhere. The code discussed in this paper is available on GitHub, and the raw data are shared with this paper.

The paper is organized as follows. In Section 2, we describe the data collection procedure used in our study. A brief overview of the movelet method by Bai et al. [14] is provided, and we also discuss variations of their method, such as alternative distance metrics and data transformations, used in our analysis. In addition, we propose a new method to quantify the uncertainty of the activity predictions from the movelet method. In Section 3, we present the results for the analysis of our study dataset. The main findings and future directions are discussed in Section 4.

## 2. Materials and Methods

### 2.1. Study Procedure

There are various publicly available datasets for performing activity recognition using smartphone sensor data (e.g., Anguita et al. [24], Micucci et al. [25], Malekzadeh et al. [26]). We conducted our own data collection because to our knowledge none of the available public datasets had all of our desired attributes: (1) collection of raw gyroscope and accelerometer data, (2) collection of data from different methods of carrying the phone (e.g., we used two smartphones in the front and back pants pockets, respectively, and reorient the front pocket phone in the four possible directions), (3) collection of wearable device data in addition to smartphone data. Our study was approved by the Harvard T.H. Chan School of Public Health Institutional Review Board in February 2018 (Protocol IRB17-2044) and the data collection took place in the summer of 2018. The eligibility requirements included being at least 18 years old and able to walk, stand, and ascend and descend stairs without assistance from a person or device. Four healthy subjects, two females and two males, enrolled in the study. We collected data on age, weight, height, sex, dominant hand, and preferred method of carrying their personal phone (e.g., pocket, hand, or bag). Table S1 in the Supplementary Materials lists the participants’ demographic characteristics. The participants ranged in age from 27 to 54. In full disclosure, Participant 1 is a co-author of this paper.

Each participant completed an approximately one-hour study visit, during which she/he was outfitted with smartphones and wearable devices. To eliminate variability due to different types of phones, all participants wore phones provided by the study for data collection. The smartphones included an iPhone in the front right pants pocket and an iPhone in the back right pants pocket. The study phones were running the Beiwe application, which is part of the open source Beiwe platform for smartphone-based digital phenotyping our group has developed. The code for the Beiwe backend is available at https://github.com/onnela-lab/beiwe-backend. The code for the Beiwe iOS and Android apps are available at https://github.com/onnela-lab/beiwe-ios and https://github.com/onnela-lab/beiwe-android, respectively. During our study, the app was configured to collect accelerometer and gyroscope data continuously at 10 Hz. Also, the participant wore ActiGraph GT9X Link wearable devices on the left wrist, right wrist, and right ankle. The ActiGraph devices collected gyroscope and accelerometer data continuously at 100 Hz. All study visits were videotaped, and the video footage were used to manually annotate each participant’s accelerometer and gyroscope datasets with ground truth activity labels at a second-by-second level. We emphasize that this paper focuses on the smartphone data only, and a joint analysis of smartphone and wearable data will be presented elsewhere.

Participants were tested separately from each other. During her/his visit, we asked the participant to perform a series of activities, which generated either training data for building the participant’s dictionary (discussed in Section 2.2) or test data for evaluating the dictionary-based classification model. During the collection of training data, the participant performed some routine activities for a short period of time. These included standing (for ≈ 10 s), walking on a flat surface (≈ 15 m), ascending a single flight of stairs, descending the flight of stairs, and performing two repetitions of chair stands (i.e., sitting down from standing, staying seated for 10 s, and standing up from sitting). During the collection of test data, the participant followed various routes on the Harvard Longwood campus that included walking, standing, sitting on benches, ascending stairs, and descending stairs. We also collected data on the participant walking at different speeds, including “normal” (their normal speed), “slow” (slower than normal), and “fast” (faster than normal). For one of the routes, we had the participant complete it four times, each time with the front pocket phone in a different orientation. In all other data collection, the front pocket phone was oriented so that it was upside down with the face of the phone against the leg and the back phone was also oriented so that it was upside down with its face against the leg. To simulate a real life setting rather than a controlled lab environment, all data were collected in public places. In addition to training and test data, we collected data from an iPhone as the participant made a call and browsed the Internet using the phone since these are common activities for phone users. An outline of the complete study protocol is given in Table 1.

The dataset used in this paper, including the accelerometer and gyroscope sub-second measurements along with activity labels, is shared on Zenodo (DOI: 10.5281/zenodo.3925679). The code for our statistical analyses is available on GitHub at https://github.com/emhuang1/augmented-movelet.

### 2.2. Movelet Method

The movelet method was proposed by Bai et al. [14] for activity classification using accelerometer data collected by a wearable device at a fixed frequency. This method only requires a small amount of training data and can be used to detect activities of the user’s choice, even those that occur only for a brief moment, such as the transition from sitting to standing. In the method, the first step is to make a list of common activities that the subject performs in their daily life, such as walking, ascending and descending stairs, standing, sitting, and running. During a clinic visit, the subject is then asked to perform each activity, and the resulting data is gathered. This data is referred to as training data, and only a few seconds of training data are required per activity. The training data are then used to build a dictionary for the subject, with each entry corresponding to a different activity. Each entry consists of “movelets,” which are defined as one-second windows of data. The movelets for a specific activity entry are obtained using the segment of training data corresponding to the activity. The data analyst slides a one-second window along this segment of data, starting with the left edge of the window at the first data point, sliding one data point at a time, until the right edge of the window meets the last data point. We will refer to movelets in the dictionary as “dictionary movelets.” The number of dictionary movelets for a given activity entry depends on the duration of training data for the activity and the frequency of data collection. For example, if there are 2 s of training data on walking collected at 10 Hz, there will be 11 dictionary movelets in the walking entry.

In our analysis in Section 3, we construct a separate dictionary for each subject using their own personal training data. Previous studies have found that using a personalized classifier model for each given subject, which could be built using their personal data (as we do in our analysis) or by leveraging data of other subjects who are similar to them, can improve classification accuracy [20,27,28].

After the collection of training data, the subject proceeds about their daily life, resulting in new data without activity labels. For the new data, the subject’s dictionary is used to make classifications of the activity occurring at any given time point. First, each movelet in the new dataset is compared to all of the dictionary movelets and the closest match is identified based on a distance metric, such as Euclidean distance. For example, if the closest match is to a dictionary movelet in the walk entry, the movelet in the new dataset is classified to be walking. Second, for a given time point, a majority vote is taken among the neighbor movelets, here taken as the movelet that begins at the time point and the movelets in the proceeding second. The majority vote determines the predicted activity label at the time point.

### 2.3. Variants of Distance Metric and Data Type

When identifying the dictionary movelet that is the closest match to a given movelet from the new data, one must use a distance metric for comparing any two movelets. Bai et al. [14] propose the following metric based on Euclidean distance. Let *n* denote the number of samples collected in one second. For any given movelet *M*, let $x=\left(x_{1},x_{2},\ldots ,x_{n}\right)$ denote the vector of x-axis data recorded over the one-second window from a given sensor (e.g., the accelerometer). These points are in order of time collected, and the time gap between adjacent points is 1/h, where *h* is the data collection frequency. Analogously, denote the *y*-axis data for movelet *M* as $y=(y_{1},y_{2},\ldots ,y_{n})$ and the *z*-axis data as $z=(z_{1},z_{2},\ldots ,z_{n})$. For any given index *i*, the values $x_{i}$, $y_{i}$, and $z_{i}$ are measured at the same time. For another movelet $M^{\prime }$, denote its data vectors as $x^{\prime }=\left(x_{1}^{\prime },x_{2}^{\prime },\ldots ,x_{n}^{\prime }\right)$ for the *x*-axis, $y^{\prime }=\left(y_{1}^{\prime },y_{2}^{\prime },\ldots ,y_{n}^{\prime }\right)$ for the *y*-axis, and $z^{\prime }=\left(z_{1}^{\prime },z_{2}^{\prime },\ldots ,z_{n}^{\prime }\right)$ for the *z*-axis. Let the function $d_{L_{2}}$ measure the Euclidean distance between two vectors of the same length, e.g.,

$$d_{L_{2}}\left(x,x^{\prime }\right)=\sqrt{\sum_{i=1}^{n}{\left(x_{i}-x_{i}^{\prime }\right)}^{2}}.$$

To compare movelets *M* and $M^{\prime }$, one can compute the Euclidean distance separately for each axis of data, yielding $d_{L_{2}}\left(x,x^{\prime }\right)$ for the *x*-axis, $d_{L_{2}}\left(y,y^{\prime }\right)$ for the *y*-axis, and $d_{L_{2}}\left(z,z^{\prime }\right)$ for the *z*-axis. The distance metric proposed by Bai et al. [14] is the average of these three axis-specific Euclidean distances.

Another distance metric can be derived using correlation The procedure is analogous to that above, except using distance metric $d_{r}$ defined as

$$d_{r}\left(x,x^{\prime }\right)=\frac{1}{n-1}\sum_{i=1}^{n}\left(\frac{x_{i}-\bar{x}}{s_{x}}\right)\left(\frac{x_{i}^{\prime }-\bar{x^{\prime }}}{s_{x^{\prime }}}\right),$$

where $\bar{x}$ and $s_{x}$ are the average and standard deviation of the observations $\left(x_{1},x_{2},\ldots ,x_{n}\right)$ and $\bar{x^{\prime }}$ and $s_{x^{\prime }}$ are the average and standard deviation of the observations $\left(x_{1}^{\prime },x_{2}^{\prime },\ldots ,x_{n}^{\prime }\right)$. When searching for the dictionary movelet that is the closest match, one would identify the dictionary movelet that maximizes the average correlation $\frac{1}{3}\left(d_{r}\left(x,x^{\prime }\right)+d_{r}\left(y,y^{\prime }\right)+d_{r}\left(z,z^{\prime }\right)\right)$. In our analysis in Section 3, our main results use the Euclidean distance metric. In addition, we present a sensitivity analysis using the correlation-based metric. A potential area of future research is to examine the performance of other distance metrics.

Another variant of the movelet method is to ignore the tri-axial data and use the vector magnitude data, defined as follows. At any given time point *t*, let $\left(x,y,z\right)$ denote the accelerometer or gyroscope data at time *t* (note that *x*, *y*, and *z* are scalar). Then the magnitude at time *t* is $\sqrt{x^{2}+y^{2}+z^{2}}$. If only magnitude data are used, each movelet is represented by a single vector of *n* points rather than three axis-specific vectors each of length *n*. The distance metric definitions are analogous to those for the tri-axial data described above, except the step of averaging axis-specific results is omitted. In our analysis in Section 3, we implement the movelet method using tri-axial data and separately using only magnitude data, and compare the results.

It should be noted that the movelet length (set in Section 2.2 as one-second long) is a tuning parameter that is chosen at the discretion of the analyst. The choice of window length can have an impact on classification results. If the length is too short, there will not be enough data to distinguish between activities. If the length is too long, windows will be more likely to contain multiple activities, making activity classification difficult. Bai et al. [14] recommended using a movelet length that would be just long enough to distinguish between different activities. Based on this guideline, they found a one-second window to be appropriate for their study. Our study included similar types of activity (e.g., walking, standing, sitting) and the same sensor sampling frequency of 10 Hz, so we also use one-second movelets in our analysis in Section 3.

### 2.4. Uncertainty Quantification

Uncertainty quantification is important for methods that make use of smartphone data since we can then identify activity classifications that are likely to be incorrect. To address this aspect, we also propose a method to quantify uncertainty in the activity classifications from the movelet method. For any given participant, consider a portion of her/his labeled data (i.e., data for which activity labels are available) that was not used to construct her/his dictionary. For example, when demonstrating this method in Section 3.6, we use a four-minute segment of labeled data, including walking, ascending stairs, descending stairs, and standing, which was not used to construct the dictionary. Using the movelet method, we compute activity label predictions for each time point in the dataset. Recall that for each time *t*, the predicted activity label is based on a majority vote—the votes are taken from the movelet beginning at time *t* in addition to neighbor movelets that begin over the subsequent second. For time *t*, define the majority vote proportion as the proportion of votes for the activity label that ultimately won the majority vote, and denote it by v(t). Define an indicator variable A(t) that indicates whether the predicted activity label at time *t* is correct. Let $\hat{l}\left(t\right)$ denote the predicted activity label at time *t*, and l(t) the true activity label at time *t*. Then we have A(t)=1 if $\hat{l}\left(t\right)=l\left(t\right)$, and A(t)=0 otherwise. We then fit a logistic regression model to the labeled four-minute dataset, using A(t) as the outcome and the majority vote proportion v(t) as the explanatory variable. Other explanatory variables can be added, such as the predicted activity label at time *t*. Based on the logistic regression model, we compute an estimated probability that the predicted label is correct. For example, if v(t) is the only explanatory variable, the estimated probability is:

$$\hat{P}\left(A\left(t\right)=1\right)=\frac{\exp\{{\hat{\beta }}_{0}+{\hat{\beta }}_{1}v\left(t\right)\}}{1+\exp\{{\hat{\beta }}_{0}+{\hat{\beta }}_{1}v\left(t\right)\}}.$$

**Figure 1:**
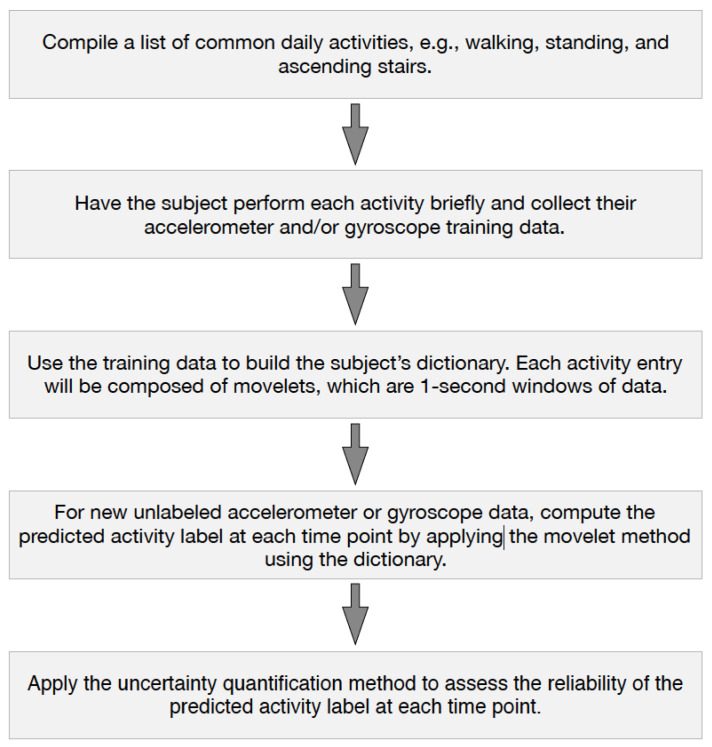
Outline of the augmented movelet method.

If this estimated probability is low, this suggests that the predicted label at time *t* is likely incorrect. We then exclude predicted labels where $\hat{P}\left(A\left(t\right)=1\right)$ is below a given threshold. We test this approach empirically and investigate the effect of the threshold choice in Section 3.6. A schematic of the augmented movelet method, including uncertainty quantification, is shown in Figure 1.

## 3. Results

### 3.1. Training Data

**Figure 2:**
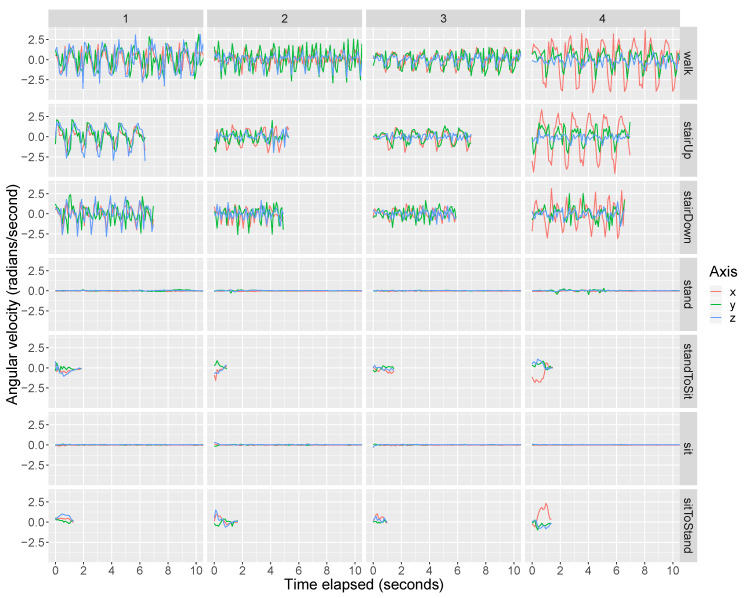
Training data (tri-axial) from the front pocket smartphone gyroscope. The raw tri-axial data (*x* = red, *y* = green, *z* = blue) from the front pocket gyroscope is shown. The columns indicate the participant ID number and the rows indicate the activity being performed. For the activities of “standToSit,” “sit,” and “sitToStand,” we plot the data from the first of the two chair-stands. In our dataset, the coordinate system for the x, y, and z axes is as follows. If you are viewing the phone by holding it in its upright position, the x-axis runs from left to right, the y-axis runs from bottom to top, and the z-axis runs through the screen towards you. To determine the direction of a rotation about any given axis, take your right hand and point your thumb in the positive direction of the axis and then curl your fingers. By doing this, your fingers will point in the direction of a positive rotation (i.e., positive angular velocity).

**Figure 3:**
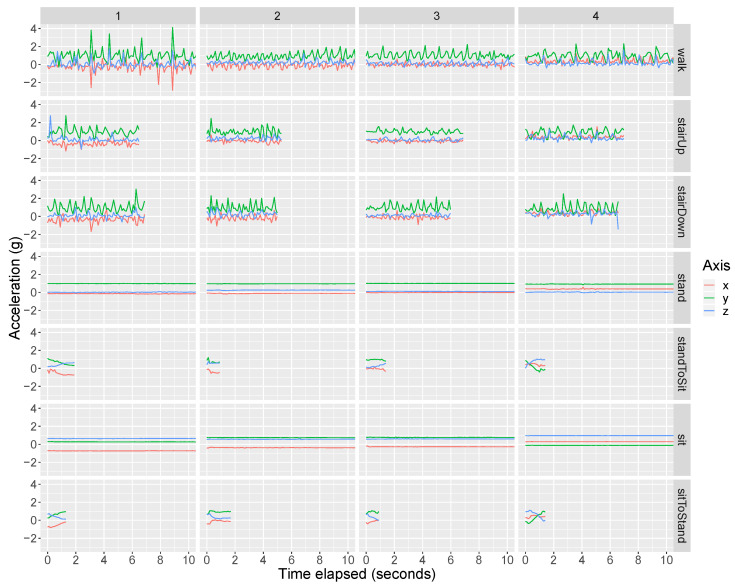
Training data (tri-axial) from the front pocket smartphone accelerometer. The raw tri-axial data (*x* = red, *y* = green, *z* = blue) from the front pocket accelerometer is shown. See Figure 2 for details.

We first present the participants’ training data. Training data were collected for standing, walking, ascending stairs, descending stairs, and chair-stands. Figure 2 and Figure 3 present the raw tri-axial (i.e., *x*, *y*, *z*) data during the training data collection for the front pocket gyroscope and accelerometer, respectively. In each of the figures, the four columns correspond to Participants 1 to 4, respectively. Each row corresponds to a specific activity. A complete chair stand was broken into three separate activities, including (i) the transition from stand-to-sit (“standToSit”), (ii) sit, and (iii) the transition from sit-to-stand (“sitToStand”). The activities “standToSit” and “sitToStand” capture the momentary transitions between sitting and standing, while “sit” captures the data occurring in between these transitions while the person was sitting. Figures S1 and S2 in the Supplementary Materials show the training data for the back pocket gyroscope and accelerometer, respectively.

In general, we observe a fair bit of variability across the participants. For example, in Figure 2, there are clear differences in the walking data across the subjects. For example, data for Participant 3 has a smaller amplitude than that for Participant 1, and for Participant 4 we see a large amplitude for the *x* axis that is not present in the data for the other participants. There is also variability between the front pocket and back pocket data. For example, for Participant 2, if we compare the front gyroscope data (Figure 2) and back gyroscope data (Figure S1 in the Supplementary Materials) during walking, the y-axis (green) has the highest amplitude in the front pocket data while the x-axis (red) has the highest amplitude in the back pocket data.

For all participants’ front and back pocket gyroscope data, the output during sitting and standing is approximately 0 radians per second because the phone is not rotating during either activity. Using the front pocket accelerometer (Figure 3), we can differentiate between sitting and standing because the phone is vertical during standing (so that we have +1g on the *y* axis) while the phone becomes closer to horizontal with potentially some tilt during sitting (so that gravity no longer falls only on the *y* axis).

We should point out that all acceleration values in our dataset include gravity. To obtain a measurement of the acceleration purely due to the subject’s movement, it is better to subtract the gravitational component. This is straightforward if the smartphone is at rest or in uniform rectilinear motion, but if the device is subject to arbitrary acceleration, supplementary measurements and adjustments, such as those based on the orientation of the device, are needed [29]. However, the way these adjustments are made likely differs across phones and phone manufacturers, and because our method is based on pattern recognition and is agnostic about the nature of acceleration, we have chosen to use unadjusted (raw) acceleration data.

Figures S3–S6 in the Supplementary Materials present the magnitude of the raw tri-axial data for the front gyroscope, back gyroscope, front accelerometer, and back accelerometer, respectively. In our results below, we apply the movelet method using tri-axial data, and perform a sensitivity analysis using magnitude data.

### 3.2. Outline of Movelet Method Application to Test Data

We applied the movelet method to accelerometer and gyroscope data separately. For each participant, we built her/his dictionary using four seconds of training data per activity. If there were more than four seconds available, we used the middle four seconds. The list of activities in the dictionary include those along the right hand margin of Figure 2. To do activity classifications for test data from the front gyroscope, we used the dictionary corresponding to the front gyroscope. The handling for the back gyroscope, front accelerometer, and back accelerometer was analogous.

We have divided the test data collection into three segments and present the results for each segment in the following three subsections. The three segments are the primary test data collection (Steps 1, 2, 5, and 6 of test data collection, see Table 1), walking at different speeds (Step 3 of test data collection), and reorienting the front pocket phone (Step 4 of test data collection).

### 3.3. Results for Primary Test Data

**Figure 4:**
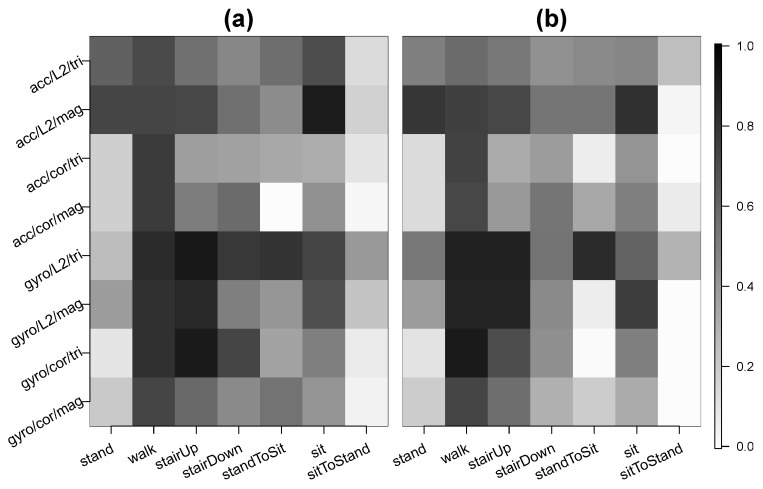
Average sensitivity across subjects for the primary test data segment (Steps 1, 2, 5, and 6). Panel (**a**) corresponds to the smartphone in the front pocket, and Panel (**b**) to the smartphone in the back pocket. For each panel, the columns are the different activities during the test data collection. Each row is a unique combination of sensor type (accelerometer or gyroscope), distance metric ($L_{2}$ distance or correlation), and data type (tri-axial or magnitude). As shown in the legend at the right, the higher the average sensitivity the darker the shading.

We first present the results for the primary test data segment. For any given activity of interest, we define a participant’s sensitivity as the proportion of predicted activity labels that are correct (i.e., that match the true activity label) when the true activity label is the activity of interest. For example, a participant’s sensitivity for walking is the proportion of predicted activity labels that are “walk” when the true activity label is “walk.” We aim for the sensitivity to be as high as possible. We computed each participant’s sensitivity for the activities of interest: standing, walking, ascending stairs, descending stairs, the transition from standing to sitting, sitting, and the transition from sitting to standing. The results were computed for each of eight unique settings of sensor type (accelerometer or gyroscope), distance metric ($L_{2}$ distance or correlation), and data type (tri-axial or magnitude). Figure 4 shows the average sensitivity across the participants for each activity of interest and under each setting. In Tables S2–S9 of the Supplementary Materials, we provide the detailed results for each participant, including their sensitivity values and their distribution of predicted activity labels for each activity. Tables S2–S5 are for the $L_{2}$ distance metric, and correspond to the front gyroscope, back gyroscope, front accelerometer, and back accelerometer, respectively. Analogously, Tables S6–S9 are for the correlation distance metric.

We first focus on the three vigorous activities: walking, ascending stairs, and descending stairs. For the accelerometer, using magnitude and $L_{2}$ distance (i.e., acc/L2/mag) tended to yield the highest average sensitivities across participants for the vigorous activities. For the front pocket data, the average sensitivity under acc/L2/mag was 0.73 for walking, 0.72 for ascending stairs, and 0.56 for descending stairs. For the back pocket data, the average sensitivity was 0.76 for walking, 0.72 for ascending stairs, and 0.54 for descending stairs. The choices acc/cor/tri and acc/cor/mag also had high average sensitivities for walking, at 0.77 and 0.78 for the front pocket and 0.74 and 0.73 for the back pocket. However, they had lower average sensitivities for the stair activities than acc/L2/mag. For example, for the front pocket, the average sensitivity for ascending stairs was 0.38 for acc/cor/tri and 0.51 for acc/cor/mag.

Comparing the two sensor types, the average sensitivities for walking, ascending stairs, and descending stairs were higher using gyroscope data than using accelerometer data. For the gyroscope, the choices of gyro/L2/tri, gyro/L2/mag, and gyro/cor/tri had the highest average sensitivities for the vigorous activities. For the front pocket, the average sensitivity for gyro/L2/tri was 0.83 for walking, 0.92 for ascending stairs, and 0.79 for descending stairs. The results for gyro/L2/mag were 0.81, 0.84, and 0.50, respectively, and the results for gyro/cor/tri were 0.81, 0.91, and 0.73. For the back pocket, the average sensitivity for gyro/L2/tri was 0.87 for walking, 0.87 for ascending stairs, and 0.55 for descending stairs. The results for gyro/L2/mag were 0.87, 0.87, and 0.46, respectively, and the results for gyro/cor/tri were 0.90, 0.70, and 0.44.

The accelerometer had higher average sensitivities than the gyroscope for the stationary activities of standing and sitting. For example, for the front pocket, the average sensitivity for standing was 0.63 for acc/L2/tri compared to 0.26 for gyro/L2/tri. Using gyroscope data, the algorithm tended to confuse standing and sitting, which is because the phone is not rotating in either case.

As shown in Figure 4, using $L_{2}$ distance as the distance metric yielded higher average sensitivities compared to using correlation in most cases. We can see this by comparing the shadings in rows 1 versus 3, 2 versus 4, 5 versus 7, and 6 versus 8 within each panel. By comparing rows 1 versus 2, 3 versus 4, etc., we can also compare the results for tri-axial data to those for magnitude data. For the accelerometer, magnitude data improved average sensitivity in most cases. For example, in the back pocket, the average sensitivity for ascending stairs was 0.53 for acc/L2/tri compared to 0.72 for acc/L2/mag. Also, the average sensitivity for sitting was 0.48 for acc/L2/tri compared to 0.81 for acc/L2/mag. For the gyroscope, using magnitude data had a higher average sensitivity in some cases and lower average sensitivity in other cases.

### 3.4. Results for Walking at Different Speeds

**Figure 5:**
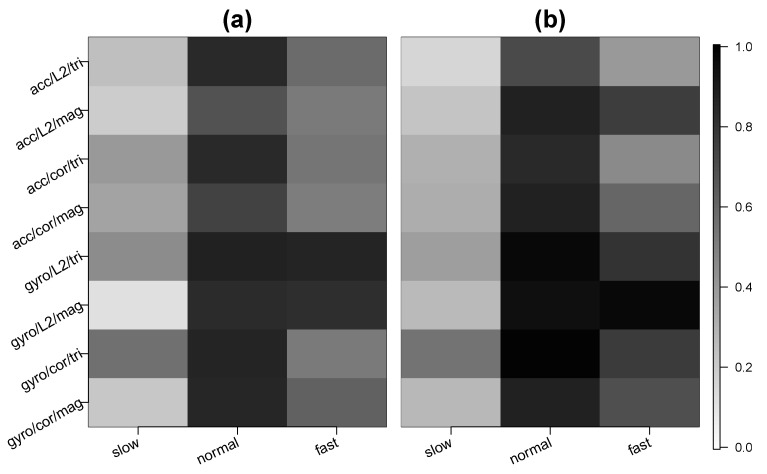
Average sensitivity across subjects for the test data collected in Step 3 (walking at different speeds). Panel (**a**) corresponds to the front pocket phone and Panel (**b**) to the back pocket phone. See Figure 4 for details.

Using the second segment of test data, we examined the case where the participant walked at different speeds (Step 3 in the Test Data section of Table 1), including a “normal,” “fast,” and “slow” speed. We examined whether the algorithm could correctly classify these activities as walking. Figure 5 shows the average sensitivity across participants separately for each walking speed, where sensitivity is defined as the proportion of predicted activity labels that are “walk.” We show the average sensitivity for each unique setting of sensor type, distance metric, and data type. In the Supplementary Materials, Tables S10 and S11 present the detailed participant-specific results when $L_{2}$ distance is used and Tables S12 and S13 present the counterpart results when the correlation distance metric is used.

The average sensitivities were highest for normal walking compared to slow and fast walking. This was expected because the training data for walking was collected at a normal speed. For normal walking, both the gyroscope and accelerometer performed well at correctly classifying walking. For the accelerometer, the average sensitivities ranged from 0.68 to 0.85 for the front pocket and from 0.71 to 0.87 for the back pocket. These ranges are from varying the distance metric and data type. The average sensitivities for the gyroscope were higher than for the accelerometer. For the gyroscope, the average sensitivities for normal walking ranged from 0.83 to 0.88 for the front pocket and from 0.87 to 0.99 for the back pocket. For each pairing of distance metric and data type, the sensitivities varied across participants. For example, using the setting of gyro/L2/tri for the front pocket, walking was correctly predicted 82% of the time for Participant 1, 100% of the time for Participant 2, 69% for Participant 3, and 100% for Participant 4, as shown in Table S10 in the Supplementary Materials.

For the gyroscope and normal walking, using magnitude data worked marginally better than using tri-axial data, as shown by the slightly darker shade for the tri-axial cells compared to their magnitude counterparts in Figure 5. For the accelerometer, interestingly, the average sensitivities tended to be lower using magnitude data than tri-axial data for the front pocket (e.g., 0.84 for acc/L2/tri versus 0.68 for acc/L2/mag), but higher for the back pocket (e.g., 0.71 for acc/L2/tri versus 0.87 for acc/L2/mag).

For fast walking, the gyroscope also had higher sensitivities than the accelerometer. The average sensitivities for the gyroscope ranged from 0.52 to 0.86 for the front pocket and from 0.69 to 0.97 for the back pocket. The average sensitivities for the accelerometer ranged from 0.51 to 0.58 for the front pocket and from 0.40 to 0.60 for the back pocket. The three highest average sensitivities for the front pocket were attained by gyro/L2/tri at 0.86, gyro/L2/mag at 0.82, and gyro/cor/mag at 0.62. For the back pocket, the highest average sensitivities were for gyro/L2/mag at 0.97, followed by gyro/L2/tri at 0.80, and then acc/L2/mag and gyro/cor/tri tied at 0.77. Thus, the best results came from using the gyroscope and $L_{2}$ distance.

For slow walking, the average sensitivities were substantially lower than for normal and fast walking. When mistaken, slow walking tended to be confused for ascending or descending stairs. The average sensitivities for the gyroscope ranged from 0.12 to 0.56 for the front pocket and from 0.27 to 0.55 for the back pocket. The average sensitivities for accelerometer ranged from 0.20 to 0.40 for the front pocket and from 0.16 to 0.32 for the back pocket. For the front pocket, the highest average sensitivity was 0.56 for gyro/cor/tri followed by gyro/L2/tri at 0.45. For the back pocket, the average sensitivity was highest again for gyro/cor/tri at 0.55, followed by gyro/L2/tri at 0.38.

### 3.5. Results for Reorienting the Front Pocket Phone

The third segment of test data collection involved reorienting the front pocket phone. A phone can be put inside a pants pocket in four potential orientations, based on whether the phone’s screen is facing the person’s leg and whether the phone is upside down or right-side-up. For each orientation, the data can be transformed back to a standard frame of reference by reversing the sign of two among the three axes (*x*,*y*,*z*) of data; which two axes depends on the orientation of the phone, as shown in Table S14 in the Supplementary Materials. However, this transformation can be difficult to utilize in practice since we may not know the orientation of the phone at every given time.

**Figure 6:**
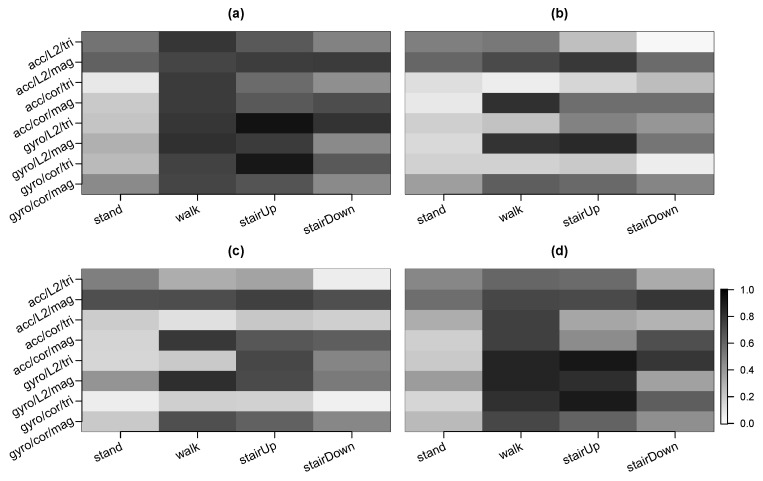
Average sensitivity across subjects for the test data collected in Step 4 (varying orientation of front pocket phone). The four panels correspond to the four potential orientations: (**a**) phone upside down with screen facing the leg; (**b**) phone upside down with screen not facing the leg; (**c**) phone right side up with screen facing the leg; (**d**) phone right side up with screen not facing the leg. Each panel gives the results for the front pocket phone. See Figure 4 for further details.

We sought to assess the movelet method’s performance when applied to data collected from another orientation compared to that used in the training data collection phase. During our test data collection, we asked the participant to repeat one course four times (Step 4 in Test Data section of Table 1). The course included standing, walking, as well as ascending and descending stairs. Each time the participant repeated the course, the phone in the front pocket was re-oriented, so that data from each possible orientation was observed. For the test data collected during this segment, we implemented the movelet method using either (i) the raw tri-axial data without any adjustment, even though the training data was collected under a single orientation, or (ii) magnitude data. We expected that using magnitude data would have better classification performance since it removes the effect of phone orientation. We also tested each of the distance metrics, $L_{2}$ distance and correlation. This analysis focused on the front pocket phone since the back pocket phone was not reoriented. Figure 6 presents the average sensitivities separately for each orientation. Participant-specific results are given in the Supplementary Materials in Tables S15–S20 for $L_{2}$ distance, and Tables S21–S26 for correlation.

Figure 6a corresponds to the orientation where the phone is upside down with its screen facing the leg. This panel has the highest average sensitivities among the four panels because it uses the same orientation as in the training data collection. Compared to Figure 6a, Figure 6d looks quite similar. For this panel, the orientation is exactly opposite that of Figure 6a, with the phone right side up and the screen not facing the leg. Although the orientation here is different from that used in the training data collection, the average sensitivities can still be high using tri-axial data, in particular for the gyroscope and the activities of walking and ascending stairs. The average sensitivities in Figure 6b,c are also similar to each other but their average sensitivities are not as high as for Figure 6a,d.

Within each panel, the rows alternate between the two data types, tri-axial and magnitude. For Figure 6b,c, the magnitude rows have improved average sensitivity (as shown by the darker shadings) compared to their tri-axial counterparts. For example, in Figure 6c, the average sensitivity for walking is 0.12 for acc/cor/tri compared to 0.78 for acc/cor/mag. For Figure 6d, using magnitude data improved average sensitivity for the accelerometer, but not for the gyroscope. For example, the average sensitivity for ascending stairs is 0.58 for acc/L2/tri compared to 0.72 for acc/L2/mag and 0.92 for gyro/L2/tri compared to 0.83 for gyro/L2/mag.

### 3.6. Uncertainty Quantification

**Figure 7:**
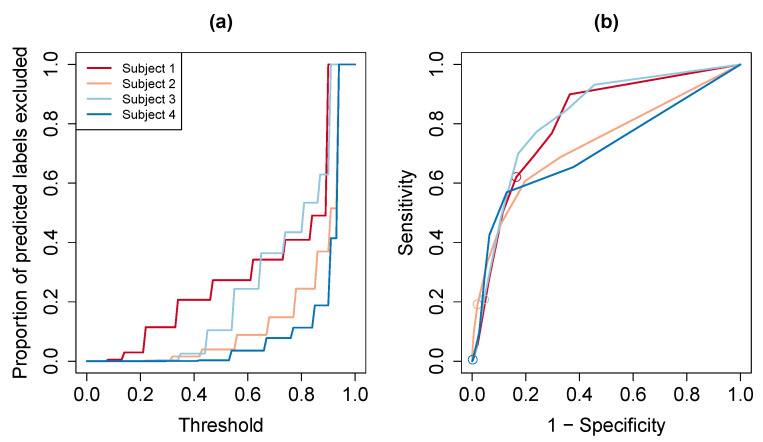
Prediction exclusions based on logistic regression model. Panel (**a**) shows the proportion of predicted labels excluded (*y*-axis) as a function of threshold choice (*x*-axis), separately for each participant. Panel (**b**) shows the participant-specific ROC curves. In each curve, we circle the point corresponding to the threshold of 0.5 as a reference.

We now present the results for the uncertainty quantification method proposed in Section 2.4. In this analysis, we implemented the movelet method using the tri-axial data and with $L_{2}$ distance, focusing on the gyroscope in the front pocket phone. The same analysis could be performed using the other variants of the movelet method (e.g., with magnitude data or the correlation distance metric). For each participant, we fitted a logistic regression model using the front gyroscope data collected when the participant performed the first step in the test data collection (see Step 1 in Test Data section of Table 1). This step was to go around the Harvard Longwood campus quadrangle, which included walking, ascending stairs, descending stairs, and standing. The explanatory variable used in the model was the majority vote proportion. We did not include the predicted activity label as another explanatory variable, since the quadrangle data did not have the sitting activities represented in the dictionary. We tested our method using the front gyroscope data collected from another time the participant went around the quadrangle with the front pocket phone in the same placement (during Step 4 in Test Data section of Table 1). For this segment of data, we used the fitted logistic regression model to estimate the probability P(A(t)=1) for every time *t*. Subsequently, if the estimated probability fell below a given threshold, the corresponding predicted activity label was excluded. To determine the effect of the threshold choice, we performed a sensitivity analysis varying the threshold from 0 to 1, at 0.01 increments. Figure 7a presents the proportion of predictions that are excluded as a function of the threshold. The higher the threshold, the more predictions are excluded. There is variability across the participants; at most thresholds, Participants 2 and 4 have less predictions excluded than Participants 1 and 3. For example, at the 0.5 threshold, the proportion of predicted labels that are excluded is 0.27 for Participant 1, 0.04 for Participant 2, 0.10 for Participant 3, and 0.003 for Participant 4. At the 0.75 threshold, the proportions excluded are 0.41, 0.15, 0.43, and 0.08, respectively.

The participant-specific ROC curves are presented in Figure 7b. Sensitivity, on the *y*-axis, represents the probability of excluding a predicted label given that the predicted label is not correct. One minus the specificity, on the *x*-axis, represents the probability of excluding a predicted label given that the predicted label is correct. Different points on each curve correspond to distinct choices for the threshold. We circle the point corresponding to the threshold of 0.5 as a reference point. At this threshold, the sensitivity/specificity pairs are 0.62/0.84 for Participant 1, 0.19/0.98 for Participant 2, 0.21/0.96 for Participant 3, and 0.01/0.998 for Participant 4. At the higher threshold of 0.75, the sensitivity/specificity pairs are 0.77/0.70 for Participant 1, 0.46/0.89 for Participant 2, 0.77/0.76 for Participant 3, and 0.28/0.95 for Participant 4. As an example of interpreting the results, for Participant 3, the proportion of incorrect labels that are excluded (sensitivity) rose from 0.21 using the 0.5-threshold to 0.77 using the 0.75-threshold. The proportion of correct labels that were not excluded (specificity) dropped from 0.96 to 0.76. As in Figure 7a, the curves differ across participants and thus the preferred threshold could also vary across participants. For example, in choosing between the 0.50 and 0.75 thresholds, we might select the 0.75 threshold for Participant 4 since 95% of correct predicted labels were retained and 28% of incorrect predicted labels were excluded. On the other hand, for Participant 1, we might prefer the 0.50 threshold because for the 0.75 threshold only 70% of correct predicted labels were retained while 84% are retained at the 0.5 threshold level.

## 4. Discussion

In this paper, we extended the movelet method to smartphone accelerometer and gyroscope data for the purpose of distinguishing between walking, ascending stairs, descending stairs, standing, and sitting. We also developed a new extension to the method for assessing the accuracy of the activity classification at each timepoint. In the analysis of our study dataset, using gyroscope data tended to outperform accelerometer data for activities that involved body movement (e.g., walking, ascending stairs, and descending stairs), while using accelerometer data was preferable for stationary activities (e.g., standing and sitting). For the activities involving movement, the method was best at correctly detecting walking and ascending stairs but had more difficulty with descending stairs. It was also better at classifying normal-paced and fast walking than slow walking, which was sometimes confused for using stairs. In our sensitivity analyses, we found that the choice of the distance metric and data type affects the activity classification results, and the optimal choice can depend on the sensor and activity types. Our method for uncertainty quantification can be used to flag activity classifications that are likely incorrect, and the appropriate threshold choice may differ from person to person.

The method as presented requires collecting training data on every participant, which can be challenging in large studies. One option is to match each participant without labeled training data to another person for whom labeled training data is available, and this matching could be based on variables, such as age, height, weight, sex, and preferred phone carrying position. A potential future area of research is to test this approach. Another way to streamline the collection of training data is to incorporate the data collection into routine tests that are already conducted during clinic visits, such as the six-minute walk test.

Another challenge is to consider different possible placements of the phone. In this paper, we focused on the case where the phone is in a pants pocket. The study data showed that the placement of the phone in the front pocket compared to the back pocket affected the data and subsequent activity classification. The data also will look different if the phone is in the hand, a backpack, or a purse. One could handle unknown and time-varying placements of the phone by augmenting the dictionary with entries for each potential method of carrying the phone, e.g., by having multiple entries for walking including “walking-pocket,” “walking-backpack,” and “walking-hand.” A tradeoff is that this will lengthen the training data collection time and increase the required computation time because there will be more movelets in the participant’s dictionary. More importantly, the number of real-life variations in carrying position may be unknown a priori, and there could be too many to incorporate into training data collection. Thus, a potential area of future research is to investigate more features, like magnitude, that are resilient to the orientation of the sensor. This type of approach would not require any expansion in the training data collection.

In addition to different placements of the phone, variability is also introduced into the smartphone data when the subject performs the same activity at a different intensity or speed. This variation that occurs naturally in free-living settings can make activity classification challenging [30], which we observed in our study when trying to classify slow walking as walking. An area of future research is making the movelet method more robust to these natural variations in smartphone data collected in free-living settings. Another potential area of future research is to preprocess the data, such as using data smoothing techniques, before implementing the movelet method and to examine the effect on classification performance.

The movelet method was designed for wearable device data that is collected continuously at a given frequency. Continuous time collection of both accelerometer and gyroscope data at 10 Hz on the smartphone results in 155,520,000 data points per subject per month, and depending on the scientific questions and other considerations (such as cost of data transfer from the phone to the study server if cellular data rather than Wi-Fi is used), relying on sampled rather than continuously collected data may be reasonable. For our smartphone-based studies, we sometimes use a data collection design that involves alternating each sensor between an on-cycle and off-cycle of prespecified lengths of time [31,32]. For the accelerometer and gyroscope, a commonly used data collection scheme is 30 s on, 30 s off. A potential area of future research is to adapt the movelet method to handle preplanned missing periods resulting from such sampling schemes.

## Figures and Tables

**Table 1:** Outline of study protocol.

	Description
Using the Phone	The participant puts on three Actigraph wearable devices, tightening the straps according to their preference. There is a wearable on the right ankle and one on each wrist. The study investigator puts a smartphone on a table. The participant picks up the phone from the table, finds the study investigator’s name under “Contacts,” calls the study investigator holding the phone to their ear, hangs up after reaching the voicemail greeting, and sets the phone back on the table. Starting with the phone on the table, the participant picks up the phone from the table, presses the icon for the Safari browser, enters the word “statistics” into the search bar, selects the Wikipedia page that appears as the first search result, scrolls through the Wikipedia entry, and sets the phone back on the table. The participant puts one smartphone in their front pants pocket and the other smartphone in their back pants pocket.
Training Data	Standing for ≈ 10 s. Walking on a flat surface for ≈ 15 m. Ascending one flight of stairs. Descending one flight of stairs. Two chair-stands (i.e., sitting down from standing, staying seated for ≈10 s, and standing up from sitting).
Test Data	Going around the Harvard Medical School Quadrangle (which consists of walking, ascending stairs, descending stairs, and standing). Walking to a bench and sitting down, then walking to another bench and sitting down. Walking at different speeds (normal, fast, then slow). Repeating (1) four times, each time with the front pocket phone in a different orientation. Walking from the Harvard Medical School Quadrangle into the Kresge building of the Harvard T.H. Chan School of Public Health (which consists of walking, ascending stairs, descending stairs, standing, and going through a revolving door to enter the building). Descending and ascending a flight of stairs in the Kresge building.

## Supplementary Materials

The following are available online at https://www.mdpi.com/1424-8220/20/13/3706/s1, Figures S1–S6, Tables S1–S26.

## Abbreviations

The following abbreviations are used in this manuscript:

acc	Accelerometer
corr	Correlation
gyro	Gyroscope
mag	Magnitude
L2	Euclidean distance
tri	Tri-axial
