# Correction: Huang, E.J., and Onnela, J.P. Augmented Movelet Method for Activity Classification Using Smartphone Gyroscope and Accelerometer Data. *Sensors* 2020, *20*(13), 3706

**DOI:** 10.3390/s20216091

**Published:** 2020-10-27

**Authors:** Emily J. Huang, Jukka-Pekka Onnela

**Affiliations:** 1 Department of Mathematics and Statistics, Wake Forest University, Winston Salem, NC 27106, USA; 2 Department of Biostatistics, Harvard T.H. Chan School of Public Health, Harvard University, Boston, MA 02115, USA; onnela@hsph.harvard.edu

The authors wish to make the following corrections to this paper [1]:

After the publication of our paper, we discovered that there was a programming error in our code. In the paper, we analyzed accelerometer and gyroscope data, and for each sensor we performed a tri-axial analysis and a magnitude analysis. Due to the programming error, our tri-axial analyses used x-axis data only, rather than the x, y, and z axes as intended. Using the x-axis data alone is a valid method, but it does not make full use of the collected data. We corrected the programming error and re-ran all of our tri-axial analyses. The tri-axial results generally improved after incorporating all three axes, compared to those reported in the paper. The average sensitivity increased from 0.48 to 0.64 for the primary test segment, when all three axes were used compared to the x-axis only. The conclusions of the paper did not change. We are very sorry for our error.

1. The authors wish to insert the following sentence in Section 2.3 at the end of Paragraph 3: “Due to a programming error, our tri-axial analyses used only the x-axis data, i.e., $d_{L_{2}}\left(x,x^{\prime }\right)$ for Euclidean distance and $d_{r}\left(x,x^{\prime }\right)$ for correlation. Although this corresponds to a valid method, it does not make full use of the collected data.”
2. The authors wish to insert the following sentence in Section 3.1 at the end of Paragraph 5: “The tri-axial results presented throughout the rest of the Results section use x-axis data only.”
