# Supplementary material for: Augmented Movelet Method for Activity Classification Using Smartphone Gyroscope and Accelerometer Data
Source: Sensors (Basel). 2020 Jul 2;20(13):3706. doi: 10.3390/s20133706 (PMC7374287; doi:10.3390/s20133706)
Supplement: Supplementary file 1 [file sensors-20-03706-s001.pdf]

Supplementary Materials for "Augmented Movelet Method for Activity Classification Using Smartphone Gyroscope and Accelerometer Data"

June 30, 2020

Table of Contents

Description of Supplemental Figures

Figures S1 and S2 show the raw tri-axial training data for the back pocket gyroscope and accelerometer, respectively. Figures S3, S4, S5, and S6 present the magnitude of the training data for the front gyroscope, back gyroscope, front accelerometer, and back accelerometer, respectively.

Description of Supplemental Tables

Participant Characteristics: Table S1 lists characteristics of the participants, including sex, weight, height, dominant hand, and preferred phone carrying position.

Primary Test Data: Tables S2, S3, S4, and S5 give the participants' results for the L_2 distance metric, and correspond to the front gyroscope, back gyroscope, front accelerometer, and back accelerometer, respectively. Tables S6, S7, S8, and S9 give the results for the correlation distance metric. Within each table, we contrast the results for tri-axial data versus magnitude data.

Changing Walking Speeds: Tables S10 and S11 present the participants' results when L_2 distance is used and Tables S12 and S13 present the counterpart results when the correlation distance metric is used. Tables S10 and S12 use tri-axial data, while Table S11 and S13 use magnitude data.

Reorienting Front Pocket Phone: Table S14 presents the procedure to transform raw tri-axial data to a standard frame of reference for each potential placement of the phone in the front pocket. Participant-specific results for the "reorienting front pocket phone" segment of the test data collection are presented in Tables S15-S20 for L_2 distance, and Tables S21-S26 for correlation. Tables S15-S16 and S21-S22 correspond to the repetition during which the phone was upside down with its screen not facing the participant's leg. Tables S17-S18 and S23-S24 correspond to the repetition during which the phone was right-side-up with its screen facing the leg. Tables S19-S20 and S25-S26 correspond to the repetition when the phone was right-side-up with the screen not facing the leg. The odd-numbered tables correspond to the front pocket gyroscope, and the even-numbered tables to the front pocket accelerometer. Within each table, we contrast the results from using tri-axial data versus those from using magnitude data.

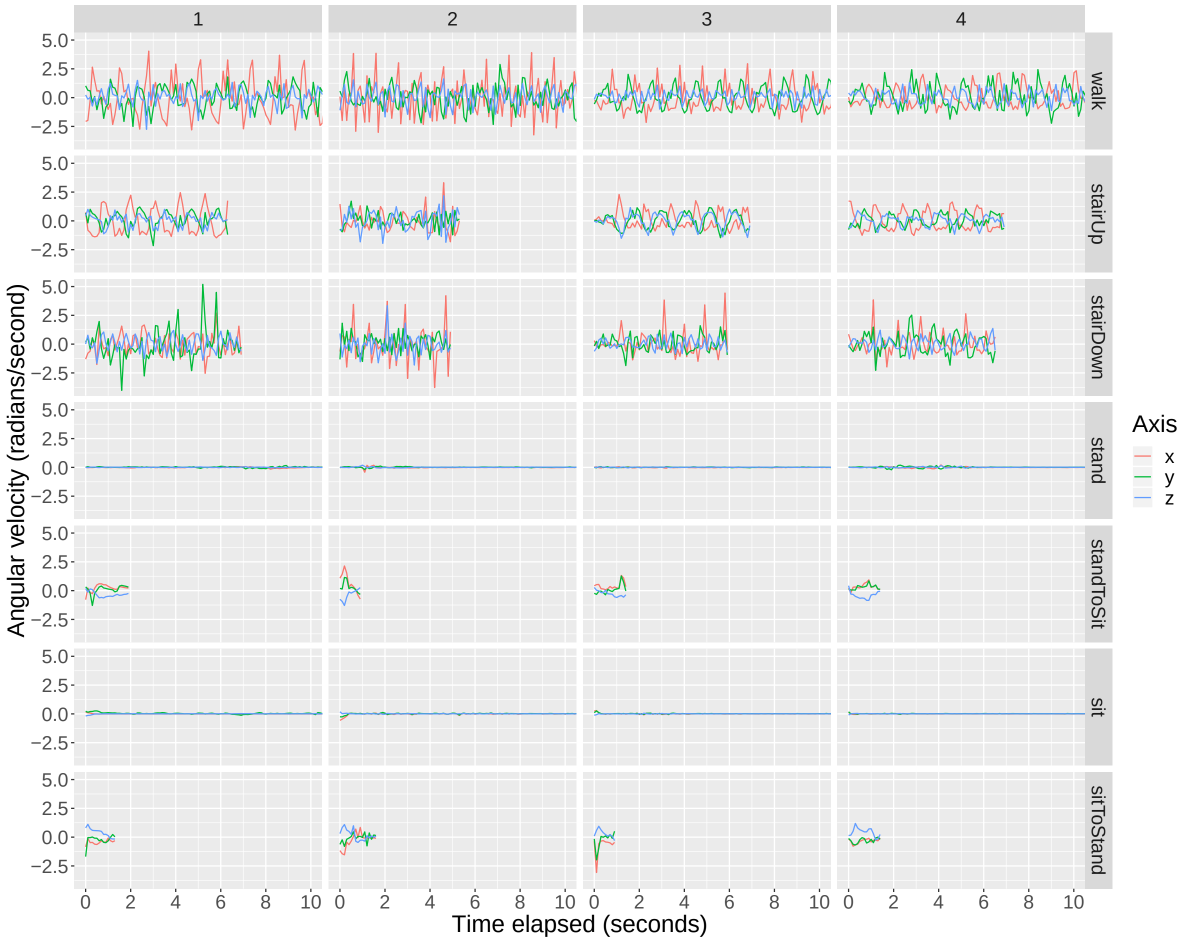

Figure S1: Training data (tri-axial) from the back pocket smartphone gyroscope. *The raw tri-axial data (x = red, y = green, z = blue) from the back pocket gyroscope is shown.*

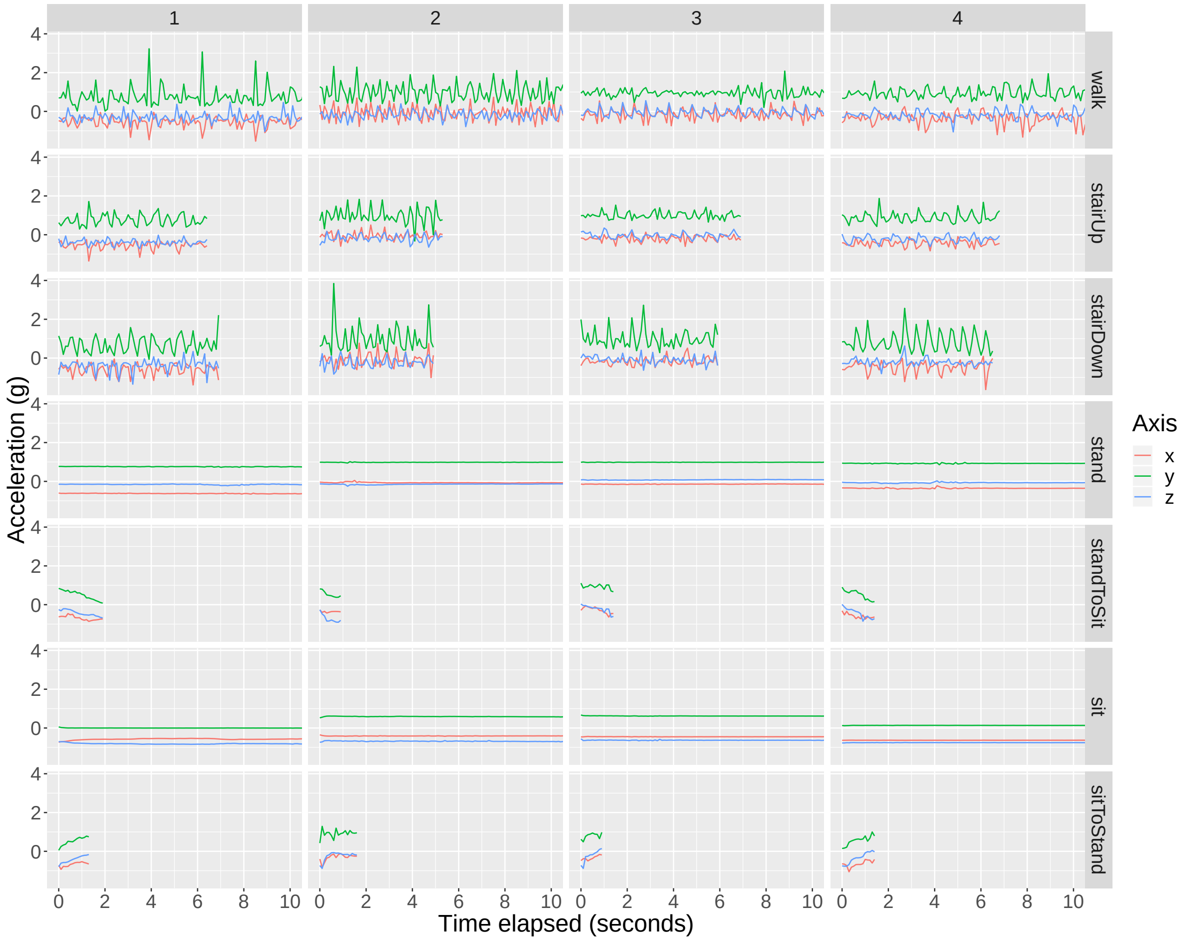

Figure S2: Training data (tri-axial) from the back pocket smartphone accelerometer. *The raw tri-axial data (x = red, y = green, z = blue) from the back pocket accelerometer is shown.*

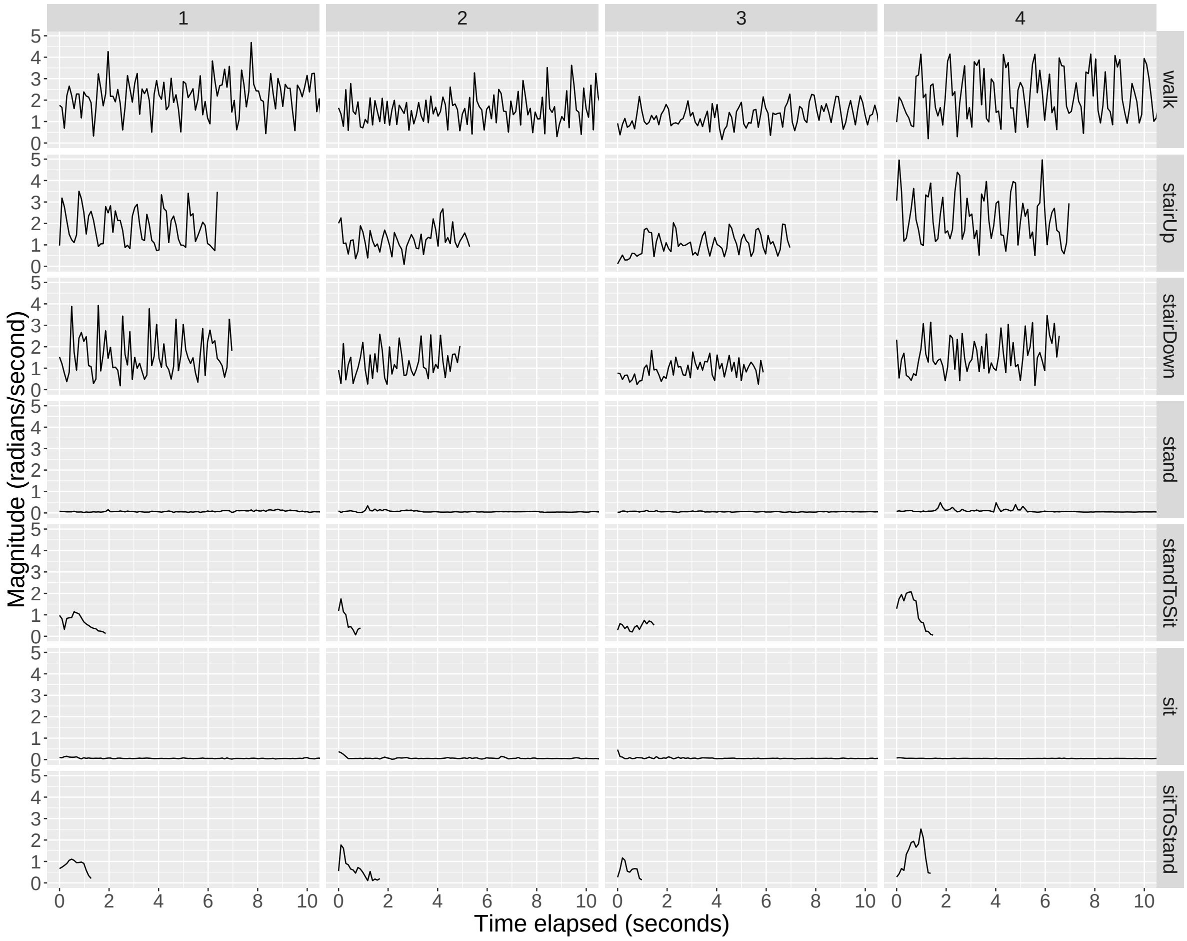

Figure S3: Training data (magnitude) from the front pocket smartphone gyroscope. *The magnitude data from the front pocket gyroscope is shown. For each time point, the magnitude is computed by squaring the measurement from each axis (yielding three quantities), summing them up, and taking the square root. The columns indicate the participant ID number and the rows indicate the activity being performed. For the activities of "standToSit," "sit," and "sitToStand," we plot the data from the first of the two chair-stands.*

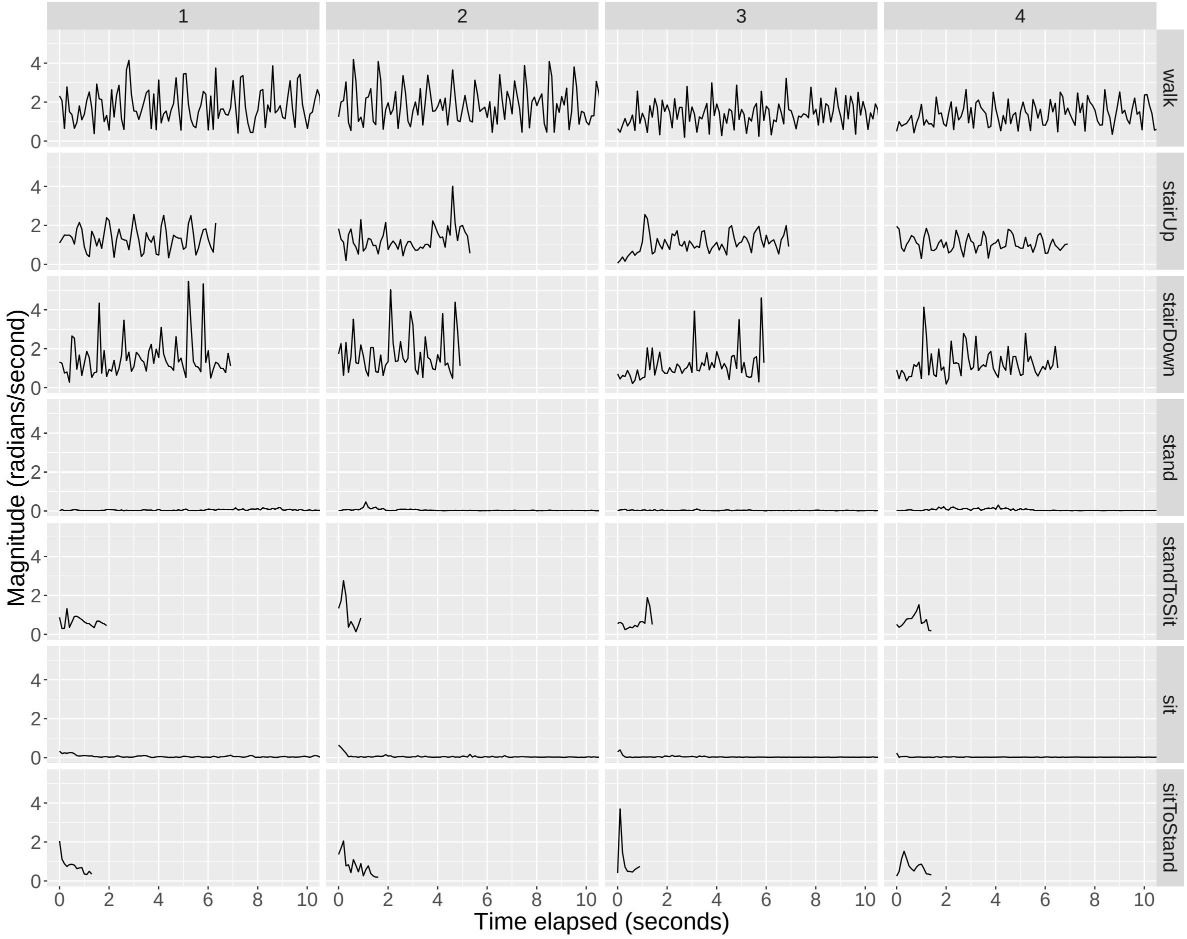

Figure S4: Training data (magnitude) from the back pocket smartphone gyroscope. *See Figure S3 for details.*

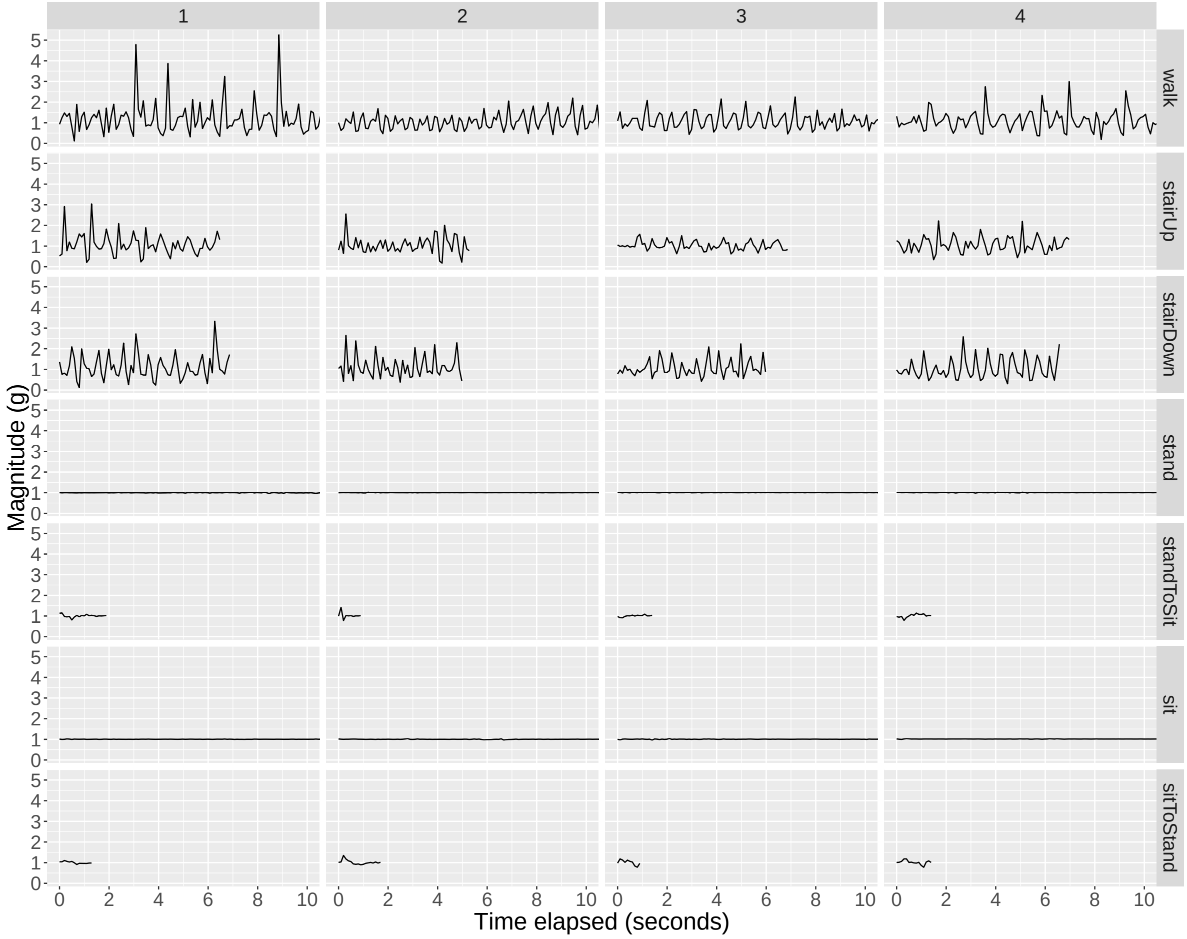

Figure S5: Training data (magnitude) from the front pocket smartphone accelerometer. *See Figure S3 for details.*

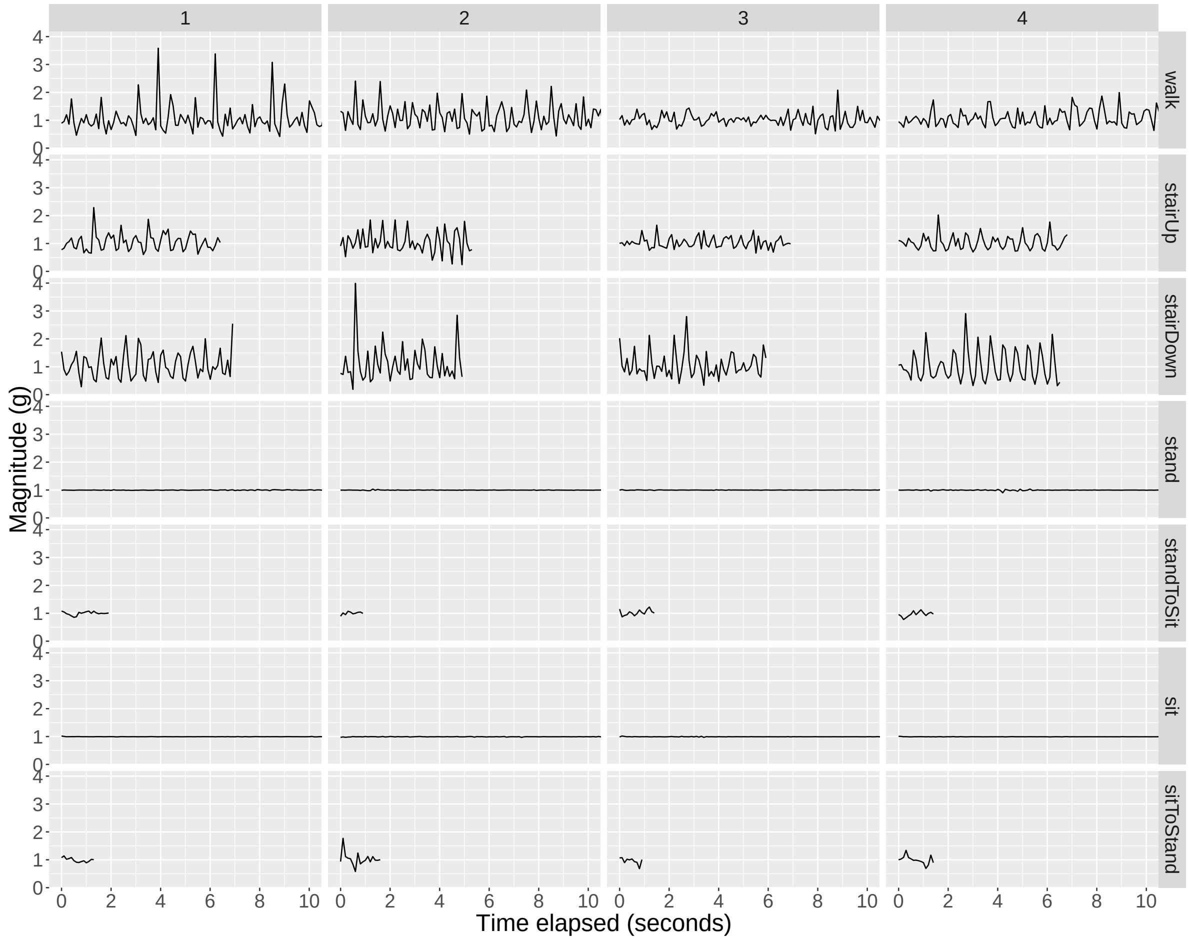

Figure S6: Training data (magnitude) from the back pocket smartphone accelerometer. *See Figure S3 for details.*

Table S1: Participant characteristics.

Participant	Sex	Height (m)	Weight (kg)	Dominant Hand	Phone Carrying Preference
1	male	1.83	86	right	pants pocket
2	female	1.75	79	right	purse, hand, pants pocket
3	female	1.60	57	right	purse, hand, pants pocket
4	male	1.88	84	right	pants pocket

Abbreviation Key: m = meters, kg = kilograms

Table S2: True activity label versus predicted activity label for front pocket smartphone gyroscope using the L_2 distance metric. *These results incorporate Steps 1, 2, 5, and 6 of the test data collection. Subtables (a)-(d) correspond to Participants 1-4, respectively. In each subtable, the column headings show the true activity label and the row headings show the predicted activity label. There are two subcolumns under each column heading; the left subcolumn is for classifications based on tri-axial data and the right subcolumn for classifications based on magnitude data. Each subcolumn gives the distribution of predicted activity labels, when the true activity label is that given by the column heading. Thus, each subcolumn sums to 1. The shaded diagonal highlights the proportion of activity label classifications that match the true activity label. The closer these diagonals are to 1 (the perfect score), the better the accuracy of the classifier. The average of the diagonal elements for each data type (tri-axial vs. magnitude) is 0.76 vs. 0.77 for Participant 1, 0.65 vs. 0.39 for Participant 2, 0.66 vs. 0.52 for Participant 3, and 0.62 vs. 0.52 for Participant 4.*

(a) Participant 1

	stand		walk		stairUp		stairDown		standToSit		sit		sitToStand	
stand	0.18	0.39	0.00	0.00	0.00	0.00	0.00	0.00	0.00	0.10	0.09	0.14	0.00	0.00
walk	0.01	0.00	0.71	0.84	0.00	0.02	0.07	0.04	0.00	0.00	0.00	0.00	0.00	0.00
stairUp	0.06	0.05	0.20	0.14	1.00	0.98	0.18	0.07	0.00	0.00	0.00	0.00	0.00	0.00
stairDown	0.04	0.00	0.07	0.01	0.00	0.00	0.76	0.86	0.00	0.00	0.00	0.00	0.05	0.00
standToSit	0.00	0.05	0.01	0.00	0.00	0.00	0.00	0.00	0.85	0.60	0.00	0.02	0.00	0.10
sit	0.70	0.48	0.00	0.00	0.00	0.00	0.00	0.00	0.15	0.00	0.86	0.81	0.00	0.00
sitToStand	0.00	0.03	0.00	0.01	0.00	0.00	0.00	0.02	0.00	0.30	0.05	0.03	0.95	0.90

(b) Participant 2

	stand		walk		stairUp		stairDown		standToSit		sit		sitToStand	
stand	0.19	0.00	0.00	0.00	0.00	0.00	0.00	0.00	0.00	0.00	0.12	0.00	0.00	0.00
walk	0.01	0.00	0.93	0.65	0.06	0.05	0.25	0.19	0.00	0.00	0.00	0.00	0.48	0.52
stairUp	0.00	0.03	0.02	0.30	0.91	0.83	0.05	0.36	0.00	0.55	0.00	0.01	0.05	0.48
stairDown	0.00	0.00	0.01	0.05	0.00	0.09	0.70	0.43	0.00	0.00	0.00	0.03	0.00	0.00
standToSit	0.00	0.01	0.02	0.00	0.00	0.02	0.00	0.02	0.60	0.00	0.00	0.06	0.00	0.00
sit	0.70	0.76	0.00	0.00	0.00	0.00	0.00	0.00	0.05	0.00	0.78	0.85	0.00	0.00
sitToStand	0.10	0.21	0.02	0.00	0.03	0.00	0.00	0.00	0.35	0.45	0.10	0.04	0.48	0.00

(c) Participant 3

	stand		walk		stairUp		stairDown		standToSit		sit		sitToStand	
stand	0.18	0.57	0.00	0.00	0.00	0.00	0.00	0.00	0.00	0.00	0.16	0.24	0.00	0.00
walk	0.00	0.00	0.73	0.84	0.00	0.17	0.10	0.16	0.00	0.25	0.00	0.02	0.10	0.95
stairUp	0.00	0.02	0.22	0.04	1.00	0.83	0.02	0.24	0.00	0.25	0.01	0.04	0.55	0.05
stairDown	0.10	0.00	0.03	0.10	0.00	0.00	0.88	0.54	0.00	0.25	0.00	0.02	0.25	0.00
standToSit	0.02	0.24	0.00	0.01	0.00	0.00	0.01	0.05	1.00	0.25	0.02	0.05	0.00	0.00
sit	0.70	0.16	0.01	0.00	0.00	0.00	0.00	0.00	0.00	0.00	0.74	0.63	0.00	0.00
sitToStand	0.00	0.00	0.00	0.00	0.00	0.00	0.00	0.01	0.00	0.00	0.06	0.00	0.10	0.00

(d) Participant 4

	stand		walk		stairUp		stairDown		standToSit		sit		sitToStand	
stand	0.49	0.59	0.01	0.00	0.00	0.00	0.00	0.00	0.25	0.20	0.35	0.40	0.00	0.00
walk	0.00	0.00	0.95	0.91	0.23	0.23	0.17	0.61	0.00	0.00	0.00	0.00	0.00	0.48
stairUp	0.00	0.00	0.01	0.05	0.76	0.73	0.02	0.21	0.00	0.00	0.00	0.00	0.00	0.10
stairDown	0.24	0.05	0.02	0.02	0.01	0.04	0.81	0.15	0.00	0.00	0.02	0.02	0.95	0.33
standToSit	0.00	0.07	0.00	0.01	0.00	0.00	0.00	0.00	0.75	0.80	0.00	0.06	0.00	0.10
sit	0.27	0.18	0.00	0.00	0.00	0.00	0.00	0.00	0.00	0.00	0.54	0.49	0.00	0.00
sitToStand	0.00	0.11	0.00	0.00	0.00	0.00	0.00	0.03	0.00	0.00	0.09	0.04	0.05	0.00

Table S3: True activity label versus predicted activity label for back pocket smartphone gyroscope using the L_2 distance metric. *See Table S2 for details. The average of the diagonal elements for each data type (tri-axial vs. magnitude) is 0.86 vs. 0.55 for Participant 1, 0.45 vs. 0.44 for Participant 2, 0.72 vs. 0.51 for Participant 3, and 0.58 vs. 0.44 for Participant 4.*

(a) Participant 1

	stand		walk		stairUp		stairDown		standToSit		sit		sitToStand	
stand	0.85	0.34	0.00	0.00	0.00	0.00	0.03	0.00	0.15	0.00	0.09	0.06	0.20	0.00
walk	0.00	0.00	0.85	0.82	0.00	0.00	0.08	0.10	0.00	0.00	0.00	0.00	0.00	0.00
stairUp	0.07	0.06	0.13	0.07	1.00	0.99	0.04	0.21	0.00	0.00	0.00	0.00	0.00	0.00
stairDown	0.00	0.04	0.00	0.11	0.00	0.01	0.83	0.67	0.00	0.00	0.00	0.00	0.00	0.00
standToSit	0.07	0.06	0.01	0.01	0.00	0.00	0.02	0.03	0.85	0.20	0.01	0.08	0.00	1.00
sit	0.00	0.52	0.00	0.00	0.00	0.00	0.00	0.00	0.00	0.05	0.86	0.85	0.00	0.00
sitToStand	0.00	0.00	0.02	0.00	0.00	0.00	0.00	0.00	0.00	0.75	0.04	0.01	0.80	0.00

(b) Participant 2

	stand		walk		stairUp		stairDown		standToSit		sit		sitToStand	
stand	0.21	0.02	0.00	0.00	0.00	0.00	0.00	0.00	0.00	0.00	0.82	0.00	0.00	0.00
walk	0.02	0.01	0.82	0.91	0.00	0.17	0.39	0.50	0.00	0.20	0.00	0.00	0.15	0.30
stairUp	0.04	0.04	0.11	0.05	0.84	0.78	0.16	0.01	0.00	0.00	0.07	0.12	0.50	0.70
stairDown	0.00	0.01	0.05	0.04	0.11	0.04	0.45	0.48	0.00	0.25	0.00	0.00	0.10	0.00
standToSit	0.00	0.00	0.01	0.00	0.00	0.00	0.00	0.00	0.80	0.00	0.00	0.00	0.25	0.00
sit	0.69	0.78	0.00	0.00	0.00	0.00	0.00	0.00	0.20	0.25	0.04	0.87	0.00	0.00
sitToStand	0.03	0.14	0.01	0.00	0.05	0.02	0.00	0.00	0.00	0.30	0.06	0.00	0.00	0.00

(c) Participant 3

	stand		walk		stairUp		stairDown		standToSit		sit		sitToStand	
stand	0.49	0.73	0.01	0.00	0.00	0.00	0.00	0.00	0.00	0.00	0.09	0.19	0.00	0.00
walk	0.00	0.00	0.92	0.94	0.01	0.08	0.26	0.41	0.00	0.45	0.00	0.00	0.25	0.60
stairUp	0.11	0.06	0.06	0.04	0.99	0.86	0.25	0.26	0.00	0.00	0.01	0.05	0.35	0.40
stairDown	0.00	0.02	0.01	0.01	0.00	0.05	0.49	0.32	0.00	0.50	0.00	0.00	0.00	0.00
standToSit	0.05	0.13	0.00	0.01	0.00	0.01	0.00	0.01	0.95	0.05	0.02	0.03	0.00	0.00
sit	0.34	0.06	0.00	0.00	0.00	0.00	0.00	0.00	0.00	0.00	0.79	0.70	0.00	0.00
sitToStand	0.00	0.00	0.00	0.00	0.00	0.00	0.00	0.00	0.05	0.00	0.08	0.02	0.40	0.00

(d) Participant 4

	stand		walk		stairUp		stairDown		standToSit		sit		sitToStand	
stand	0.55	0.48	0.00	0.00	0.00	0.00	0.05	0.00	0.26	0.11	0.14	0.18	0.25	0.00
walk	0.00	0.04	0.91	0.81	0.34	0.05	0.38	0.41	0.00	0.00	0.00	0.00	0.00	0.30
stairUp	0.00	0.04	0.06	0.13	0.66	0.84	0.06	0.21	0.00	0.74	0.00	0.02	0.70	0.50
stairDown	0.04	0.03	0.01	0.05	0.00	0.11	0.44	0.36	0.00	0.00	0.00	0.00	0.05	0.00
standToSit	0.09	0.07	0.00	0.01	0.00	0.00	0.03	0.02	0.74	0.00	0.00	0.10	0.00	0.20
sit	0.33	0.28	0.00	0.00	0.00	0.00	0.00	0.00	0.00	0.00	0.74	0.61	0.00	0.00
sitToStand	0.00	0.08	0.01	0.00	0.00	0.00	0.05	0.00	0.00	0.16	0.12	0.08	0.00	0.00

Table S4: True activity label versus predicted activity label for front pocket smartphone accelerometer using the L_2 distance metric. *See Table S2 for details. The average of the diagonal elements for each data type (tri-axial vs. magnitude) is 0.66 vs. 0.72 for Participant 1, 0.52 vs. 0.56 for Participant 2, 0.54 vs. 0.67 for Participant 3, and 0.45 vs. 0.48 for Participant 4.*

(a) Participant 1

	stand		walk		stairUp		stairDown		standToSit		sit		sitToStand	
stand	0.91	0.85	0.00	0.00	0.00	0.00	0.01	0.00	0.00	0.00	0.00	0.00	0.00	0.33
walk	0.01	0.00	0.64	0.62	0.00	0.07	0.05	0.12	0.00	0.00	0.00	0.00	0.00	0.00
stairUp	0.07	0.03	0.18	0.26	0.72	0.91	0.52	0.10	0.00	0.00	0.00	0.00	0.33	0.24
stairDown	0.00	0.00	0.18	0.10	0.26	0.00	0.41	0.78	0.00	0.00	0.00	0.00	0.14	0.00
standToSit	0.00	0.04	0.00	0.03	0.01	0.02	0.00	0.00	0.55	0.85	0.00	0.02	0.00	0.29
sit	0.00	0.08	0.00	0.00	0.00	0.00	0.00	0.00	0.45	0.15	0.99	0.96	0.14	0.05
sitToStand	0.00	0.00	0.00	0.00	0.00	0.00	0.00	0.00	0.00	0.00	0.01	0.01	0.38	0.10

(b) Participant 2

	stand		walk		stairUp		stairDown		standToSit		sit		sitToStand	
stand	1.00	0.63	0.00	0.00	0.18	0.00	0.00	0.00	0.00	0.00	0.01	0.00	0.20	0.00
walk	0.00	0.00	0.80	0.92	0.18	0.43	0.32	0.40	0.00	0.00	0.00	0.00	0.30	0.05
stairUp	0.00	0.00	0.02	0.06	0.28	0.33	0.07	0.00	0.00	0.00	0.00	0.00	0.50	0.30
stairDown	0.00	0.00	0.10	0.00	0.22	0.07	0.53	0.51	0.00	0.00	0.00	0.00	0.00	0.00
standToSit	0.00	0.01	0.01	0.00	0.00	0.02	0.00	0.05	1.00	0.00	0.94	0.00	0.00	0.00
sit	0.00	0.37	0.07	0.01	0.10	0.00	0.08	0.00	0.00	0.95	0.00	0.94	0.00	0.05
sitToStand	0.00	0.00	0.00	0.01	0.04	0.15	0.00	0.03	0.00	0.05	0.06	0.06	0.00	0.60

(c) Participant 3

	stand		walk		stairUp		stairDown		standToSit		sit		sitToStand	
stand	0.55	0.69	0.00	0.00	0.00	0.00	0.00	0.00	0.00	0.00	0.00	0.00	0.05	0.00
walk	0.00	0.01	0.57	0.62	0.03	0.01	0.07	0.27	0.05	0.00	0.00	0.00	0.30	0.30
stairUp	0.41	0.02	0.30	0.20	0.86	0.97	0.49	0.17	0.35	0.05	0.02	0.02	0.50	0.65
stairDown	0.01	0.04	0.13	0.15	0.06	0.00	0.36	0.56	0.00	0.00	0.00	0.00	0.00	0.00
standToSit	0.00	0.05	0.00	0.02	0.04	0.01	0.00	0.00	0.30	0.95	0.00	0.10	0.00	0.00
sit	0.03	0.19	0.00	0.00	0.01	0.00	0.07	0.00	0.30	0.00	0.96	0.88	0.00	0.05
sitToStand	0.00	0.00	0.00	0.00	0.00	0.00	0.00	0.00	0.00	0.00	0.02	0.00	0.15	0.00

(d) Participant 4

	stand		walk		stairUp		stairDown		standToSit		sit		sitToStand	
stand	0.04	0.79	0.00	0.00	0.00	0.00	0.00	0.00	0.25	0.45	0.03	0.10	0.00	0.00
walk	0.00	0.00	0.86	0.77	0.08	0.27	0.21	0.40	0.10	0.00	0.00	0.00	0.30	0.15
stairUp	0.00	0.07	0.03	0.20	0.40	0.66	0.17	0.22	0.00	0.00	0.00	0.00	0.00	0.50
stairDown	0.09	0.08	0.07	0.01	0.29	0.05	0.56	0.38	0.00	0.00	0.00	0.00	0.00	0.00
standToSit	0.00	0.00	0.00	0.01	0.00	0.02	0.00	0.00	0.45	0.00	0.01	0.07	0.20	0.35
sit	0.87	0.01	0.04	0.00	0.23	0.00	0.07	0.00	0.20	0.05	0.88	0.77	0.50	0.00
sitToStand	0.00	0.04	0.00	0.01	0.00	0.00	0.00	0.00	0.00	0.50	0.08	0.06	0.00	0.00

Table S5: True activity label versus predicted activity label for back pocket smartphone accelerometer using the L_2 distance metric. *See Table S2 for details. The average of the diagonal elements for each data type (tri-axial vs. magnitude) is 0.73 vs. 0.71 for Participant 1, 0.07 vs. 0.55 for Participant 2, 0.66 vs. 0.58 for Participant 3, and 0.40 vs. 0.57 for Participant 4.*

(a) Participant 1

	stand		walk		stairUp		stairDown		standToSit		sit		sitToStand	
stand	0.93	0.69	0.01	0.00	0.03	0.00	0.00	0.00	0.00	0.10	0.00	0.11	0.05	0.00
walk	0.00	0.00	0.71	0.76	0.03	0.02	0.21	0.07	0.00	0.00	0.00	0.00	0.00	0.20
stairUp	0.00	0.01	0.21	0.18	0.73	0.95	0.14	0.28	0.00	0.00	0.00	0.00	0.00	0.00
stairDown	0.00	0.00	0.06	0.03	0.10	0.00	0.62	0.65	0.00	0.00	0.00	0.00	0.00	0.00
standToSit	0.00	0.06	0.01	0.03	0.00	0.03	0.01	0.00	0.60	0.90	0.06	0.04	0.00	0.50
sit	0.06	0.24	0.00	0.00	0.10	0.00	0.01	0.00	0.00	0.00	0.91	0.85	0.35	0.15
sitToStand	0.01	0.00	0.01	0.00	0.01	0.00	0.00	0.00	0.40	0.00	0.03	0.00	0.60	0.15

(b) Participant 2

	stand		walk		stairUp		stairDown		standToSit		sit		sitToStand	
stand	0.00	0.80	0.00	0.00	0.00	0.00	0.00	0.00	0.05	0.00	0.51	0.05	0.00	0.00
walk	0.00	0.04	0.36	0.93	0.03	0.14	0.15	0.49	0.00	0.20	0.00	0.00	0.00	0.15
stairUp	0.00	0.00	0.02	0.02	0.00	0.39	0.10	0.06	0.00	0.15	0.00	0.02	0.00	0.60
stairDown	0.00	0.00	0.04	0.01	0.00	0.28	0.06	0.46	0.00	0.00	0.00	0.00	0.00	0.00
standToSit	0.02	0.00	0.14	0.03	0.04	0.13	0.07	0.00	0.00	0.40	0.07	0.07	1.00	0.25
sit	0.25	0.16	0.35	0.00	0.68	0.04	0.53	0.00	0.15	0.25	0.03	0.86	0.00	0.00
sitToStand	0.73	0.00	0.09	0.01	0.24	0.02	0.08	0.00	0.80	0.00	0.38	0.00	0.00	0.00

(c) Participant 3

	stand		walk		stairUp		stairDown		standToSit		sit		sitToStand	
stand	0.84	0.87	0.00	0.00	0.01	0.00	0.04	0.00	0.00	0.15	0.00	0.09	0.00	0.00
walk	0.00	0.03	0.67	0.64	0.04	0.04	0.18	0.20	0.00	0.00	0.00	0.00	0.00	0.05
stairUp	0.01	0.03	0.24	0.13	0.71	0.92	0.42	0.26	0.00	0.55	0.00	0.01	0.55	0.75
stairDown	0.10	0.04	0.08	0.19	0.22	0.02	0.36	0.49	0.00	0.00	0.00	0.00	0.05	0.00
standToSit	0.05	0.03	0.01	0.03	0.01	0.02	0.00	0.05	0.65	0.30	0.00	0.04	0.00	0.20
sit	0.00	0.00	0.00	0.01	0.00	0.00	0.00	0.00	0.35	0.00	0.98	0.84	0.00	0.00
sitToStand	0.00	0.00	0.00	0.00	0.00	0.00	0.00	0.00	0.00	0.00	0.02	0.02	0.40	0.00

(d) Participant 4

	stand		walk		stairUp		stairDown		standToSit		sit		sitToStand	
stand	0.24	0.82	0.00	0.00	0.00	0.01	0.00	0.00	0.20	0.05	0.87	0.22	0.00	0.00
walk	0.00	0.00	0.58	0.70	0.09	0.21	0.23	0.38	0.20	0.40	0.00	0.00	0.00	0.40
stairUp	0.01	0.01	0.31	0.27	0.68	0.62	0.08	0.04	0.00	0.00	0.00	0.00	0.00	0.40
stairDown	0.07	0.10	0.08	0.00	0.16	0.11	0.69	0.58	0.00	0.00	0.00	0.00	0.15	0.00
standToSit	0.14	0.03	0.03	0.03	0.05	0.06	0.00	0.00	0.60	0.55	0.06	0.04	0.85	0.20
sit	0.52	0.04	0.00	0.00	0.00	0.00	0.00	0.00	0.00	0.00	0.00	0.69	0.00	0.00
sitToStand	0.01	0.00	0.00	0.00	0.02	0.00	0.00	0.00	0.00	0.00	0.08	0.05	0.00	0.00

Table S6: True activity label versus predicted activity label for front pocket smartphone gyroscope using the correlation distance metric. *See Table S2 for details. The average of the diagonal elements for each data type (tri-axial vs. magnitude) is 0.64 vs. 0.59 for Participant 1, 0.41 vs. 0.32 for Participant 2, 0.43 vs. 0.34 for Participant 3, and 0.50 vs. 0.47 for Participant 4.*

(a) Participant 1

	stand		walk		stairUp		stairDown		standToSit		sit		sitToStand	
stand	0.06	0.16	0.00	0.01	0.00	0.00	0.00	0.00	0.00	0.00	0.06	0.20	0.00	0.00
walk	0.11	0.08	0.80	0.74	0.00	0.03	0.08	0.15	0.00	0.00	0.04	0.16	0.00	0.33
stairUp	0.15	0.07	0.16	0.10	1.00	0.88	0.05	0.02	0.00	0.00	0.10	0.04	0.43	0.00
stairDown	0.22	0.07	0.03	0.07	0.00	0.00	0.84	0.77	0.00	0.00	0.06	0.04	0.19	0.00
standToSit	0.12	0.06	0.00	0.00	0.00	0.00	0.00	0.00	1.00	0.95	0.24	0.02	0.05	0.48
sit	0.33	0.55	0.01	0.08	0.00	0.09	0.02	0.05	0.00	0.05	0.51	0.46	0.05	0.00
sitToStand	0.01	0.00	0.01	0.00	0.00	0.00	0.00	0.00	0.00	0.00	0.00	0.07	0.29	0.19

(b) Participant 2

	stand		walk		stairUp		stairDown		standToSit		sit		sitToStand	
stand	0.05	0.18	0.01	0.03	0.00	0.13	0.00	0.04	0.00	0.00	0.00	0.05	0.00	0.00
walk	0.06	0.06	0.76	0.65	0.03	0.08	0.18	0.12	0.00	0.00	0.06	0.21	0.14	0.52
stairUp	0.52	0.00	0.03	0.06	0.90	0.13	0.07	0.03	0.85	0.00	0.13	0.16	0.48	0.00
stairDown	0.04	0.03	0.02	0.10	0.00	0.16	0.70	0.45	0.05	0.00	0.28	0.03	0.00	0.00
standToSit	0.07	0.25	0.00	0.01	0.00	0.00	0.03	0.00	0.00	0.40	0.00	0.07	0.00	0.00
sit	0.22	0.44	0.17	0.15	0.07	0.48	0.02	0.37	0.10	0.50	0.50	0.42	0.38	0.48
sitToStand	0.06	0.04	0.00	0.00	0.00	0.01	0.00	0.00	0.00	0.10	0.03	0.06	0.00	0.00

(c) Participant 3

	stand		walk		stairUp		stairDown		standToSit		sit		sitToStand	
stand	0.10	0.33	0.00	0.05	0.00	0.05	0.02	0.03	0.05	0.50	0.07	0.35	0.00	0.80
walk	0.12	0.06	0.72	0.63	0.00	0.17	0.14	0.08	0.00	0.00	0.01	0.01	0.00	0.00
stairUp	0.23	0.23	0.24	0.05	0.98	0.75	0.09	0.08	0.45	0.15	0.30	0.22	1.00	0.00
stairDown	0.06	0.00	0.00	0.10	0.01	0.01	0.62	0.36	0.10	0.25	0.06	0.09	0.00	0.20
standToSit	0.00	0.01	0.00	0.01	0.00	0.01	0.00	0.03	0.00	0.00	0.00	0.02	0.00	0.00
sit	0.48	0.37	0.04	0.15	0.01	0.01	0.13	0.43	0.40	0.10	0.56	0.31	0.00	0.00
sitToStand	0.00	0.00	0.00	0.01	0.00	0.00	0.00	0.00	0.00	0.00	0.00	0.00	0.00	0.00

(d) Participant 4

	stand		walk		stairUp		stairDown		standToSit		sit		sitToStand	
stand	0.15	0.18	0.00	0.01	0.00	0.07	0.00	0.19	0.45	0.10	0.21	0.15	0.00	0.00
walk	0.08	0.05	0.96	0.92	0.22	0.16	0.15	0.18	0.00	0.00	0.06	0.08	0.10	0.00
stairUp	0.25	0.04	0.02	0.02	0.74	0.61	0.00	0.00	0.10	0.00	0.18	0.11	0.52	0.00
stairDown	0.21	0.11	0.01	0.02	0.03	0.07	0.78	0.26	0.00	0.00	0.11	0.05	0.38	0.33
standToSit	0.06	0.00	0.00	0.00	0.00	0.00	0.00	0.00	0.45	0.85	0.02	0.04	0.00	0.00
sit	0.24	0.61	0.00	0.03	0.00	0.09	0.06	0.37	0.00	0.05	0.42	0.47	0.00	0.67
sitToStand	0.00	0.00	0.00	0.00	0.00	0.00	0.00	0.00	0.00	0.00	0.00	0.09	0.00	0.00

Table S7: True activity label versus predicted activity label for back pocket smartphone gyroscope using the correlation distance metric. See Table S2 for details. The average of the diagonal elements for each data type (tri-axial vs. magnitude) is 0.43 vs. 0.41 for Participant 1, 0.31 vs. 0.28 for Participant 2, 0.43 vs. 0.30 for Participant 3, and 0.36 vs. 0.34 for Participant 4.

(a) Participant 1

	stand		walk		stairUp		stairDown		standToSit		sit		sitToStand	
stand	0.09	0.10	0.01	0.04	0.00	0.01	0.03	0.12	0.10	0.00	0.09	0.18	0.00	0.00
walk	0.21	0.12	0.90	0.71	0.01	0.01	0.20	0.09	0.25	0.00	0.05	0.07	0.10	0.15
stairUp	0.33	0.11	0.08	0.10	0.99	0.88	0.00	0.15	0.40	0.10	0.14	0.11	0.90	0.00
stairDown	0.01	0.00	0.00	0.04	0.00	0.00	0.42	0.50	0.20	0.00	0.15	0.11	0.00	0.35
standToSit	0.07	0.09	0.01	0.01	0.00	0.00	0.00	0.00	0.05	0.35	0.02	0.19	0.00	0.25
sit	0.29	0.58	0.01	0.10	0.00	0.10	0.36	0.14	0.00	0.05	0.55	0.32	0.00	0.25
sitToStand	0.00	0.00	0.00	0.00	0.00	0.00	0.00	0.00	0.00	0.50	0.00	0.02	0.00	0.00

(b) Participant 2

	stand		walk		stairUp		stairDown		standToSit		sit		sitToStand	
stand	0.11	0.43	0.01	0.03	0.07	0.19	0.08	0.08	0.00	0.00	0.10	0.11	0.00	0.00
walk	0.09	0.00	0.82	0.57	0.08	0.14	0.36	0.29	0.00	0.00	0.14	0.22	0.05	0.20
stairUp	0.26	0.07	0.03	0.06	0.38	0.21	0.05	0.03	0.00	0.20	0.12	0.22	0.00	0.00
stairDown	0.05	0.15	0.05	0.10	0.09	0.21	0.49	0.35	0.00	0.00	0.26	0.03	0.55	0.00
standToSit	0.00	0.00	0.00	0.00	0.02	0.00	0.00	0.00	0.00	0.00	0.00	0.02	0.00	0.00
sit	0.41	0.29	0.09	0.25	0.36	0.25	0.03	0.25	0.90	0.80	0.37	0.41	0.40	0.80
sitToStand	0.09	0.05	0.00	0.00	0.00	0.01	0.00	0.00	0.10	0.00	0.00	0.00	0.00	0.00

(c) Participant 3

	stand		walk		stairUp		stairDown		standToSit		sit		sitToStand	
stand	0.10	0.08	0.01	0.01	0.01	0.07	0.04	0.14	0.10	0.50	0.11	0.15	0.35	0.00
walk	0.10	0.00	0.96	0.89	0.12	0.06	0.36	0.40	0.25	0.00	0.09	0.09	0.65	0.00
stairUp	0.27	0.35	0.03	0.04	0.84	0.73	0.03	0.08	0.25	0.00	0.13	0.24	0.00	0.15
stairDown	0.16	0.11	0.01	0.02	0.02	0.06	0.50	0.19	0.00	0.50	0.08	0.21	0.00	0.00
standToSit	0.10	0.21	0.00	0.01	0.00	0.01	0.03	0.02	0.00	0.00	0.00	0.05	0.00	0.00
sit	0.26	0.26	0.00	0.04	0.01	0.06	0.04	0.17	0.40	0.00	0.58	0.24	0.00	0.85
sitToStand	0.00	0.00	0.00	0.00	0.00	0.00	0.00	0.00	0.00	0.00	0.00	0.02	0.00	0.00

(d) Participant 4

	stand		walk		stairUp		stairDown		standToSit		sit		sitToStand	
stand	0.13	0.16	0.00	0.03	0.04	0.01	0.19	0.19	0.00	0.11	0.09	0.09	0.00	0.00
walk	0.13	0.06	0.94	0.76	0.32	0.14	0.19	0.08	0.00	0.00	0.09	0.08	0.15	0.00
stairUp	0.28	0.06	0.03	0.10	0.61	0.47	0.00	0.19	0.95	0.00	0.17	0.16	0.85	0.00
stairDown	0.06	0.07	0.01	0.06	0.00	0.07	0.36	0.19	0.00	0.00	0.11	0.05	0.00	0.00
standToSit	0.01	0.39	0.00	0.00	0.01	0.01	0.03	0.02	0.00	0.42	0.00	0.24	0.00	0.75
sit	0.35	0.27	0.01	0.04	0.02	0.29	0.24	0.33	0.00	0.47	0.51	0.35	0.00	0.25
sitToStand	0.04	0.00	0.00	0.00	0.00	0.01	0.00	0.00	0.05	0.00	0.02	0.01	0.00	0.00

Table S8: True activity label versus predicted activity label for front pocket smartphone accelerometer using the correlation distance metric. See Table S2 for details. The average of the diagonal elements for each data type (tri-axial vs. magnitude) is 0.47 vs. 0.37 for Participant 1, 0.34 vs. 0.33 for Participant 2, 0.32 vs. 0.38 for Participant 3, and 0.28 vs. 0.35 for Participant 4.

(a) Participant 1

	stand		walk		stairUp		stairDown		standToSit		sit		sitToStand	
stand	0.12	0.09	0.02	0.03	0.03	0.00	0.08	0.00	0.05	0.00	0.18	0.12	0.00	0.00
walk	0.06	0.24	0.74	0.75	0.30	0.29	0.34	0.19	0.35	1.00	0.14	0.23	0.00	0.10
stairUp	0.06	0.07	0.03	0.04	0.50	0.57	0.07	0.03	0.00	0.00	0.03	0.02	0.29	0.00
stairDown	0.07	0.13	0.12	0.05	0.07	0.00	0.41	0.73	0.00	0.00	0.08	0.07	0.00	0.00
standToSit	0.13	0.02	0.00	0.00	0.00	0.01	0.00	0.00	0.60	0.00	0.02	0.06	0.10	0.38
sit	0.54	0.44	0.09	0.14	0.10	0.13	0.10	0.05	0.00	0.00	0.52	0.48	0.24	0.52
sitToStand	0.02	0.00	0.00	0.00	0.00	0.00	0.00	0.00	0.00	0.00	0.02	0.02	0.38	0.00

(b) Participant 2

	stand		walk		stairUp		stairDown		standToSit		sit		sitToStand	
stand	0.29	0.23	0.07	0.00	0.04	0.28	0.04	0.00	0.00	0.00	0.25	0.19	0.00	0.45
walk	0.08	0.01	0.72	0.89	0.17	0.24	0.15	0.37	0.00	0.10	0.18	0.15	0.40	0.05
stairUp	0.11	0.25	0.07	0.02	0.48	0.23	0.22	0.04	0.00	0.35	0.14	0.13	0.60	0.00
stairDown	0.05	0.06	0.03	0.01	0.16	0.10	0.46	0.53	0.25	0.05	0.26	0.10	0.00	0.00
standToSit	0.29	0.03	0.00	0.00	0.00	0.05	0.00	0.04	0.30	0.00	0.00	0.03	0.00	0.00
sit	0.14	0.33	0.10	0.08	0.15	0.05	0.13	0.02	0.20	0.50	0.10	0.32	0.00	0.40
sitToStand	0.05	0.09	0.00	0.00	0.00	0.05	0.00	0.00	0.25	0.00	0.07	0.08	0.00	0.10

(c) Participant 3

	stand		walk		stairUp		stairDown		standToSit		sit		sitToStand	
stand	0.22	0.18	0.00	0.02	0.05	0.03	0.08	0.02	0.00	0.05	0.10	0.20	0.20	0.00
walk	0.07	0.24	0.79	0.63	0.15	0.03	0.09	0.16	0.05	0.30	0.17	0.04	0.20	0.40
stairUp	0.13	0.19	0.07	0.10	0.21	0.67	0.21	0.00	0.45	0.35	0.11	0.07	0.35	0.00
stairDown	0.07	0.21	0.10	0.18	0.17	0.11	0.44	0.57	0.20	0.00	0.26	0.04	0.05	0.45
standToSit	0.01	0.00	0.00	0.00	0.04	0.00	0.00	0.01	0.30	0.00	0.07	0.04	0.00	0.00
sit	0.50	0.18	0.03	0.07	0.39	0.16	0.18	0.24	0.00	0.30	0.27	0.61	0.20	0.15
sitToStand	0.00	0.00	0.00	0.00	0.00	0.00	0.00	0.00	0.00	0.00	0.02	0.00	0.00	0.00

(d) Participant 4

	stand		walk		stairUp		stairDown		standToSit		sit		sitToStand	
stand	0.12	0.26	0.02	0.02	0.08	0.05	0.15	0.25	0.00	0.50	0.14	0.23	0.20	0.25
walk	0.02	0.23	0.83	0.84	0.14	0.23	0.16	0.07	0.00	0.00	0.14	0.17	0.05	0.00
stairUp	0.21	0.09	0.02	0.12	0.32	0.59	0.09	0.08	0.25	0.35	0.12	0.10	0.00	0.15
stairDown	0.11	0.17	0.01	0.02	0.00	0.09	0.17	0.48	0.00	0.15	0.08	0.09	0.00	0.25
standToSit	0.01	0.00	0.00	0.00	0.00	0.00	0.00	0.00	0.15	0.00	0.06	0.07	0.30	0.00
sit	0.46	0.24	0.12	0.00	0.46	0.05	0.43	0.12	0.60	0.00	0.36	0.32	0.45	0.35
sitToStand	0.05	0.00	0.00	0.00	0.00	0.00	0.00	0.00	0.00	0.00	0.10	0.02	0.00	0.00

Table S9: True activity label versus predicted activity label for back pocket smartphone accelerometer using the correlation distance metric. See Table S2 for details. The average of the diagonal elements for each data type (tri-axial vs. magnitude) is 0.42 vs. 0.51 for Participant 1, 0.20 vs. 0.32 for Participant 2, 0.24 vs. 0.30 for Participant 3, and 0.33 vs. 0.41 for Participant 4.

(a) Participant 1

	stand		walk		stairUp		stairDown		standToSit		sit		sitToStand	
stand	0.23	0.18	0.04	0.01	0.04	0.00	0.03	0.00	0.00	0.00	0.01	0.17	0.00	0.00
walk	0.29	0.17	0.81	0.85	0.22	0.07	0.35	0.18	0.20	0.20	0.04	0.19	0.05	0.20
stairUp	0.02	0.20	0.10	0.04	0.58	0.61	0.01	0.06	0.10	0.00	0.00	0.02	0.20	0.00
stairDown	0.10	0.03	0.05	0.05	0.16	0.25	0.60	0.73	0.00	0.00	0.03	0.09	0.00	0.10
standToSit	0.05	0.03	0.00	0.00	0.00	0.00	0.00	0.00	0.05	0.45	0.23	0.09	0.00	0.40
sit	0.31	0.41	0.00	0.04	0.00	0.07	0.00	0.04	0.65	0.35	0.68	0.44	0.75	0.00
sitToStand	0.00	0.00	0.00	0.00	0.00	0.00	0.00	0.00	0.00	0.00	0.01	0.01	0.00	0.30

(b) Participant 2

	stand		walk		stairUp		stairDown		standToSit		sit		sitToStand	
stand	0.10	0.03	0.08	0.01	0.22	0.11	0.03	0.04	0.00	0.00	0.13	0.10	0.35	0.50
walk	0.07	0.13	0.65	0.75	0.20	0.18	0.42	0.48	0.00	0.00	0.09	0.17	0.20	0.20
stairUp	0.09	0.08	0.14	0.02	0.18	0.23	0.18	0.02	0.00	0.45	0.22	0.08	0.05	0.25
stairDown	0.07	0.09	0.07	0.02	0.22	0.37	0.20	0.39	0.00	0.20	0.12	0.09	0.05	0.00
standToSit	0.13	0.05	0.00	0.00	0.00	0.00	0.00	0.00	0.00	0.30	0.13	0.01	0.00	0.00
sit	0.45	0.54	0.05	0.21	0.15	0.11	0.18	0.04	0.90	0.05	0.27	0.56	0.35	0.05
sitToStand	0.10	0.09	0.00	0.00	0.02	0.00	0.00	0.04	0.10	0.00	0.03	0.00	0.00	0.00

(c) Participant 3

	stand		walk		stairUp		stairDown		standToSit		sit		sitToStand	
stand	0.10	0.22	0.02	0.02	0.00	0.21	0.04	0.06	0.00	0.10	0.19	0.08	0.00	0.15
walk	0.12	0.08	0.71	0.58	0.17	0.03	0.34	0.06	0.00	0.00	0.06	0.11	0.50	0.00
stairUp	0.08	0.08	0.11	0.03	0.30	0.35	0.14	0.07	0.00	0.10	0.04	0.21	0.50	0.35
stairDown	0.18	0.15	0.14	0.25	0.04	0.16	0.24	0.57	0.00	0.55	0.23	0.15	0.00	0.20
standToSit	0.12	0.00	0.01	0.00	0.00	0.00	0.01	0.02	0.20	0.00	0.21	0.03	0.00	0.25
sit	0.32	0.47	0.03	0.12	0.49	0.25	0.23	0.21	0.80	0.25	0.10	0.42	0.00	0.05
sitToStand	0.08	0.01	0.00	0.00	0.00	0.00	0.00	0.00	0.00	0.00	0.16	0.00	0.00	0.00

(d) Participant 4

	stand		walk		stairUp		stairDown		standToSit		sit		sitToStand	
stand	0.12	0.12	0.02	0.08	0.13	0.02	0.01	0.06	0.00	0.00	0.07	0.14	0.10	0.15
walk	0.14	0.17	0.80	0.73	0.12	0.32	0.35	0.19	0.00	0.00	0.06	0.09	0.00	0.20
stairUp	0.17	0.04	0.11	0.11	0.26	0.39	0.09	0.07	0.00	0.00	0.02	0.06	0.50	0.00
stairDown	0.12	0.07	0.02	0.01	0.26	0.14	0.51	0.48	0.00	0.00	0.05	0.00	0.40	0.00
standToSit	0.14	0.00	0.00	0.01	0.00	0.05	0.00	0.07	0.00	0.60	0.07	0.13	0.00	0.50
sit	0.33	0.60	0.04	0.05	0.21	0.08	0.04	0.13	1.00	0.40	0.63	0.57	0.00	0.15
sitToStand	0.00	0.00	0.00	0.00	0.02	0.00	0.00	0.00	0.00	0.00	0.10	0.02	0.00	0.00

Table S10: Distribution of predicted activity label for each walking speed, when using tri-axial data and L_2 distance metric. *These results are for Step 3 in the test data collection, during which the participant walked at different speeds. In each subtable, the first column heading indicates the walking speed (Slow, Normal, Fast). The second heading indicates the sensor type, either accelerometer ("Acc") or gyroscope ("Gyro"). The third heading indicates whether the sensor was in the front or back pocket phone. The rows are the predicted activity labels. Each column of values shows the distribution of the predicted activity labels and sums to 1. The shaded row highlights the proportion of labels correctly predicted as walking. The average of the shaded elements for each data type (Acc-front, Acc-back, Gyro-front, Gyro-back) is (0.49, 0.77, 0.69, 0.91) for Participant 1, (0.72, 0.32, 0.87, 0.53) for Participant 2, (0.48, 0.33, 0.56, 0.71) for Participant 3, and (0.54, 0.27, 0.80, 0.73) for Participant 4.*

(a) Participant 1

	Slow				Normal				Fast			
	Acc		Gyro		Acc		Gyro		Acc		Gyro	
	front	back	front	back	front	back	front	back	front	back	front	back
stand	0.01	0.00	0.00	0.00	0.02	0.00	0.00	0.00	0.00	0.00	0.00	0.00
walk	0.59	0.59	0.55	0.80	0.59	0.88	0.82	0.93	0.30	0.84	0.71	1.00
stairUp	0.29	0.29	0.45	0.18	0.17	0.06	0.17	0.07	0.21	0.08	0.11	0.00
stairDown	0.11	0.08	0.00	0.02	0.21	0.05	0.01	0.00	0.49	0.08	0.18	0.00
standToSit	0.00	0.00	0.00	0.00	0.00	0.00	0.00	0.00	0.00	0.00	0.00	0.00
sit	0.00	0.03	0.00	0.00	0.00	0.00	0.00	0.00	0.00	0.00	0.00	0.00
sitToStand	0.00	0.00	0.00	0.00	0.00	0.00	0.00	0.00	0.00	0.00	0.00	0.00

(b) Participant 2

	Slow				Normal				Fast			
	Acc		Gyro		Acc		Gyro		Acc		Gyro	
	front	back	front	back	front	back	front	back	front	back	front	back
stand	0.39	0.00	0.00	0.00	0.00	0.00	0.00	0.00	0.00	0.00	0.00	0.00
walk	0.24	0.00	0.60	0.02	1.00	0.67	1.00	1.00	0.91	0.30	1.00	0.58
stairUp	0.18	0.00	0.00	0.37	0.00	0.00	0.00	0.00	0.01	0.23	0.00	0.04
stairDown	0.18	0.00	0.00	0.50	0.00	0.08	0.00	0.00	0.08	0.00	0.00	0.38
standToSit	0.00	1.00	0.00	0.05	0.00	0.24	0.00	0.00	0.00	0.44	0.00	0.00
sit	0.00	0.00	0.03	0.00	0.00	0.00	0.00	0.00	0.00	0.00	0.00	0.00
sitToStand	0.00	0.00	0.36	0.06	0.00	0.02	0.00	0.00	0.00	0.02	0.00	0.00

(c) Participant 3

	Slow				Normal				Fast			
	Acc		Gyro		Acc		Gyro		Acc		Gyro	
	front	back	front	back	front	back	front	back	front	back	front	back
stand	0.00	0.00	0.00	0.00	0.00	0.00	0.00	0.00	0.00	0.00	0.00	0.00
walk	0.11	0.00	0.23	0.22	0.86	0.74	0.69	0.98	0.47	0.23	0.75	0.93
stairUp	0.85	0.85	0.72	0.78	0.12	0.21	0.22	0.02	0.51	0.11	0.23	0.01
stairDown	0.04	0.01	0.05	0.00	0.02	0.03	0.08	0.00	0.01	0.66	0.03	0.06
standToSit	0.00	0.02	0.00	0.00	0.00	0.02	0.00	0.00	0.00	0.00	0.00	0.00
sit	0.00	0.00	0.00	0.00	0.00	0.00	0.00	0.00	0.00	0.00	0.00	0.00
sitToStand	0.00	0.12	0.00	0.00	0.00	0.00	0.00	0.00	0.00	0.00	0.00	0.00

(d) Participant 4

	Slow				Normal				Fast			
	Acc		Gyro		Acc		Gyro		Acc		Gyro	
	front	back	front	back	front	back	front	back	front	back	front	back
stand	0.00	0.00	0.00	0.04	0.00	0.00	0.00	0.00	0.00	0.00	0.00	0.00
walk	0.06	0.04	0.41	0.49	0.91	0.55	1.00	1.00	0.66	0.24	1.00	0.69
stairUp	0.05	0.68	0.02	0.46	0.01	0.42	0.00	0.00	0.02	0.40	0.00	0.31
stairDown	0.21	0.24	0.56	0.00	0.08	0.03	0.00	0.00	0.32	0.36	0.00	0.00
standToSit	0.00	0.02	0.01	0.00	0.00	0.00	0.00	0.00	0.00	0.00	0.00	0.00
sit	0.69	0.00	0.00	0.00	0.01	0.00	0.00	0.00	0.00	0.00	0.00	0.00
sitToStand	0.00	0.02	0.00	0.01	0.00	0.00	0.00	0.00	0.00	0.00	0.00	0.00

Table S11: Distribution of predicted activity label for each walking speed, when using magnitude data and L_2 distance metric. *See Table S10 for details. The average of the shaded elements for each data-type (Acc-front, Acc-back, Gyro-front, Gyro-back) is (0.38, 0.77, 0.72, 0.81) for Participant 1, (0.68, 0.60, 0.64, 0.83) for Participant 2, (0.34, 0.55, 0.48, 0.64) for Participant 3, and (0.47, 0.57, 0.53, 0.62) for Participant 4.*

(a) Participant 1

	Slow				Normal				Fast			
	Acc		Gyro		Acc		Gyro		Acc		Gyro	
	front	back	front	back	front	back	front	back	front	back	front	back
stand	0.00	0.00	0.00	0.00	0.00	0.00	0.00	0.00	0.00	0.00	0.00	0.00
walk	0.27	0.76	0.22	0.57	0.52	0.73	0.94	0.94	0.34	0.82	1.00	0.94
stairUp	0.67	0.10	0.76	0.22	0.26	0.23	0.03	0.00	0.12	0.05	0.00	0.00
stairDown	0.03	0.03	0.02	0.21	0.21	0.03	0.00	0.06	0.54	0.12	0.00	0.06
standToSit	0.02	0.09	0.00	0.00	0.00	0.00	0.00	0.00	0.00	0.00	0.00	0.00
sit	0.00	0.00	0.00	0.00	0.00	0.00	0.00	0.00	0.00	0.00	0.00	0.00
sitToStand	0.01	0.02	0.00	0.00	0.00	0.00	0.02	0.00	0.00	0.00	0.00	0.00

(b) Participant 2

	Slow				Normal				Fast			
	Acc		Gyro		Acc		Gyro		Acc		Gyro	
	front	back	front	back	front	back	front	back	front	back	front	back
stand	0.00	0.00	0.00	0.00	0.00	0.00	0.00	0.00	0.00	0.00	0.00	0.00
walk	0.15	0.08	0.00	0.49	1.00	1.00	0.92	1.00	0.89	0.74	1.00	1.00
stairUp	0.83	0.26	0.98	0.51	0.00	0.00	0.08	0.00	0.01	0.16	0.00	0.00
stairDown	0.00	0.00	0.00	0.00	0.00	0.00	0.00	0.00	0.05	0.10	0.00	0.00
standToSit	0.00	0.43	0.00	0.00	0.00	0.00	0.00	0.00	0.05	0.00	0.00	0.00
sit	0.02	0.00	0.00	0.00	0.00	0.00	0.00	0.00	0.00	0.00	0.00	0.00
sitToStand	0.00	0.24	0.02	0.00	0.00	0.00	0.00	0.00	0.00	0.00	0.00	0.00

(c) Participant 3

	Slow				Normal				Fast			
	Acc		Gyro		Acc		Gyro		Acc		Gyro	
	front	back	front	back	front	back	front	back	front	back	front	back
stand	0.00	0.00	0.00	0.00	0.00	0.00	0.00	0.00	0.00	0.00	0.00	0.00
walk	0.06	0.06	0.00	0.00	0.56	0.83	0.47	0.93	0.39	0.75	0.96	1.00
stairUp	0.71	0.66	0.70	0.91	0.34	0.03	0.05	0.07	0.09	0.11	0.00	0.00
stairDown	0.00	0.07	0.23	0.09	0.10	0.10	0.48	0.00	0.52	0.11	0.04	0.00
standToSit	0.20	0.20	0.07	0.00	0.00	0.00	0.00	0.00	0.00	0.03	0.00	0.00
sit	0.00	0.01	0.00	0.00	0.00	0.00	0.00	0.00	0.00	0.00	0.00	0.00
sitToStand	0.02	0.00	0.00	0.00	0.00	0.03	0.00	0.00	0.00	0.00	0.00	0.00

(d) Participant 4

	Slow				Normal				Fast			
	Acc		Gyro		Acc		Gyro		Acc		Gyro	
	front	back	front	back	front	back	front	back	front	back	front	back
stand	0.00	0.33	0.00	0.00	0.00	0.00	0.00	0.00	0.00	0.00	0.00	0.00
walk	0.30	0.03	0.25	0.01	0.65	0.92	0.99	0.91	0.45	0.76	0.34	0.95
stairUp	0.16	0.26	0.16	0.65	0.35	0.08	0.01	0.06	0.39	0.14	0.66	0.00
stairDown	0.00	0.00	0.46	0.04	0.00	0.00	0.00	0.03	0.16	0.10	0.00	0.05
standToSit	0.36	0.37	0.10	0.15	0.00	0.01	0.00	0.00	0.00	0.00	0.00	0.00
sit	0.00	0.00	0.00	0.00	0.00	0.00	0.00	0.00	0.00	0.00	0.00	0.00
sitToStand	0.18	0.00	0.02	0.15	0.00	0.00	0.00	0.00	0.00	0.00	0.00	0.00

Table S12: Distribution of predicted activity label for each walking speed, when using tri-axial data and correlation distance metric. *See Table S10 for details. The average of the shaded elements for each data-type (Acc-front, Acc-back, Gyro-front, Gyro-back) is (0.68, 0.85, 0.76, 0.95) for Participant 1, (0.50, 0.52, 0.41, 0.52) for Participant 2, (0.65, 0.40, 0.54, 0.91) for Participant 3, and (0.55, 0.39, 0.88, 0.70) for Participant 4.*

(a) Participant 1

	Slow				Normal				Fast			
	Acc		Gyro		Acc		Gyro		Acc		Gyro	
	front	back	front	back	front	back	front	back	front	back	front	back
stand	0.03	0.15	0.00	0.00	0.02	0.00	0.00	0.00	0.00	0.00	0.00	0.00
walk	0.91	0.70	0.75	0.98	0.73	0.92	0.86	0.97	0.42	0.94	0.66	0.91
stairUp	0.06	0.11	0.20	0.01	0.00	0.07	0.14	0.03	0.01	0.06	0.21	0.05
stairDown	0.01	0.02	0.02	0.00	0.13	0.00	0.00	0.00	0.24	0.00	0.07	0.00
standToSit	0.00	0.00	0.00	0.01	0.00	0.00	0.00	0.00	0.03	0.00	0.00	0.05
sit	0.00	0.02	0.04	0.00	0.11	0.01	0.00	0.00	0.30	0.00	0.06	0.00
sitToStand	0.00	0.01	0.00	0.00	0.00	0.00	0.00	0.00	0.00	0.00	0.00	0.00

(b) Participant 2

	Slow				Normal				Fast			
	Acc		Gyro		Acc		Gyro		Acc		Gyro	
	front	back	front	back	front	back	front	back	front	back	front	back
stand	0.13	0.25	0.00	0.00	0.13	0.00	0.00	0.00	0.02	0.10	0.00	0.00
walk	0.17	0.11	0.26	0.03	0.76	0.97	0.87	1.00	0.57	0.48	0.11	0.52
stairUp	0.47	0.42	0.20	0.00	0.10	0.03	0.00	0.00	0.18	0.09	0.00	0.00
stairDown	0.07	0.13	0.21	0.63	0.00	0.00	0.00	0.00	0.08	0.04	0.01	0.32
standToSit	0.00	0.00	0.00	0.00	0.00	0.00	0.00	0.00	0.00	0.00	0.00	0.00
sit	0.16	0.10	0.33	0.34	0.00	0.00	0.13	0.00	0.15	0.29	0.88	0.16
sitToStand	0.00	0.00	0.00	0.00	0.00	0.00	0.00	0.00	0.00	0.00	0.00	0.00

(c) Participant 3

	Slow				Normal				Fast			
	Acc		Gyro		Acc		Gyro		Acc		Gyro	
	front	back	front	back	front	back	front	back	front	back	front	back
stand	0.04	0.00	0.00	0.00	0.00	0.00	0.00	0.00	0.12	0.26	0.16	0.00
walk	0.38	0.28	0.61	0.83	0.95	0.75	0.71	1.00	0.63	0.16	0.31	0.91
stairUp	0.25	0.54	0.37	0.17	0.05	0.08	0.29	0.00	0.16	0.02	0.49	0.00
stairDown	0.23	0.05	0.00	0.00	0.00	0.10	0.00	0.00	0.00	0.54	0.01	0.09
standToSit	0.00	0.00	0.00	0.00	0.00	0.00	0.00	0.00	0.00	0.00	0.00	0.00
sit	0.10	0.12	0.02	0.00	0.00	0.06	0.00	0.00	0.09	0.03	0.04	0.00
sitToStand	0.00	0.00	0.00	0.00	0.00	0.00	0.00	0.00	0.00	0.00	0.00	0.00

(d) Participant 4

	Slow				Normal				Fast			
	Acc		Gyro		Acc		Gyro		Acc		Gyro	
	front	back	front	back	front	back	front	back	front	back	front	back
stand	0.02	0.05	0.01	0.07	0.00	0.01	0.00	0.00	0.07	0.13	0.00	0.00
walk	0.16	0.15	0.64	0.35	0.95	0.74	1.00	1.00	0.56	0.26	1.00	0.74
stairUp	0.21	0.33	0.31	0.52	0.00	0.18	0.00	0.00	0.01	0.30	0.00	0.00
stairDown	0.00	0.36	0.02	0.00	0.00	0.00	0.00	0.00	0.04	0.11	0.00	0.00
standToSit	0.00	0.00	0.00	0.02	0.01	0.00	0.00	0.00	0.00	0.00	0.00	0.00
sit	0.62	0.11	0.02	0.03	0.04	0.08	0.00	0.00	0.33	0.20	0.00	0.26
sitToStand	0.00	0.00	0.00	0.00	0.00	0.00	0.00	0.00	0.00	0.00	0.00	0.00

Table S13: Distribution of predicted activity label for each walking speed, when using magnitude data and correlation distance metric. See Table S10 for details. The average of the shaded elements for each data-type (Acc-front, Acc-back, Gyro-front, Gyro-back) is (0.64, 0.80, 0.64, 0.67) for Participant 1, (0.69, 0.55, 0.61, 0.62) for Participant 2, (0.36, 0.48, 0.29, 0.53) for Participant 3, and (0.46, 0.57, 0.72, 0.63) for Participant 4.

(a) Participant 1

	Slow				Normal				Fast			
	Acc		Gyro		Acc		Gyro		Acc		Gyro	
	front	back	front	back	front	back	front	back	front	back	front	back
stand	0.03	0.00	0.02	0.09	0.05	0.00	0.00	0.00	0.00	0.00	0.02	0.00
walk	0.72	0.98	0.22	0.39	0.69	0.90	0.89	0.91	0.51	0.53	0.80	0.70
stairUp	0.09	0.00	0.32	0.20	0.01	0.08	0.00	0.00	0.01	0.14	0.00	0.00
stairDown	0.00	0.00	0.06	0.06	0.14	0.02	0.05	0.03	0.21	0.18	0.18	0.10
standToSit	0.00	0.00	0.00	0.00	0.00	0.00	0.00	0.00	0.00	0.00	0.00	0.00
sit	0.16	0.02	0.38	0.27	0.10	0.00	0.05	0.06	0.27	0.15	0.00	0.20
sitToStand	0.00	0.00	0.00	0.00	0.00	0.00	0.00	0.00	0.00	0.00	0.00	0.00

(b) Participant 2

	Slow				Normal				Fast			
	Acc		Gyro		Acc		Gyro		Acc		Gyro	
	front	back	front	back	front	back	front	back	front	back	front	back
stand	0.04	0.02	0.02	0.01	0.00	0.00	0.05	0.05	0.00	0.22	0.00	0.00
walk	0.58	0.17	0.00	0.41	0.99	0.86	0.91	0.74	0.51	0.62	0.91	0.71
stairUp	0.07	0.13	0.35	0.20	0.01	0.00	0.00	0.11	0.00	0.04	0.00	0.19
stairDown	0.07	0.13	0.01	0.18	0.00	0.00	0.04	0.00	0.11	0.08	0.00	0.00
standToSit	0.00	0.00	0.00	0.00	0.00	0.00	0.00	0.00	0.09	0.00	0.00	0.00
sit	0.24	0.56	0.61	0.19	0.00	0.14	0.00	0.11	0.30	0.04	0.09	0.10
sitToStand	0.00	0.00	0.01	0.02	0.00	0.00	0.00	0.00	0.00	0.00	0.00	0.00

(c) Participant 3

	Slow				Normal				Fast			
	Acc		Gyro		Acc		Gyro		Acc		Gyro	
	front	back	front	back	front	back	front	back	front	back	front	back
stand	0.02	0.00	0.10	0.00	0.00	0.01	0.07	0.00	0.00	0.03	0.02	0.00
walk	0.06	0.07	0.17	0.02	0.58	0.83	0.70	0.96	0.45	0.54	0.00	0.60
stairUp	0.61	0.10	0.26	0.54	0.11	0.03	0.00	0.00	0.00	0.05	0.00	0.00
stairDown	0.18	0.33	0.07	0.14	0.24	0.07	0.08	0.04	0.45	0.27	0.25	0.00
standToSit	0.00	0.06	0.02	0.00	0.00	0.02	0.02	0.00	0.00	0.05	0.00	0.01
sit	0.13	0.44	0.37	0.30	0.07	0.05	0.11	0.00	0.10	0.06	0.73	0.38
sitToStand	0.00	0.00	0.00	0.00	0.00	0.00	0.02	0.00	0.00	0.00	0.00	0.00

(d) Participant 4

	Slow				Normal				Fast			
	Acc		Gyro		Acc		Gyro		Acc		Gyro	
	front	back	front	back	front	back	front	back	front	back	front	back
stand	0.46	0.18	0.06	0.12	0.07	0.09	0.00	0.00	0.00	0.05	0.00	0.00
walk	0.10	0.07	0.46	0.27	0.71	0.90	0.92	0.88	0.57	0.73	0.78	0.74
stairUp	0.21	0.00	0.17	0.11	0.22	0.00	0.00	0.03	0.39	0.08	0.13	0.17
stairDown	0.01	0.12	0.00	0.25	0.00	0.00	0.00	0.05	0.01	0.14	0.04	0.04
standToSit	0.00	0.02	0.00	0.05	0.00	0.00	0.00	0.04	0.00	0.00	0.00	0.00
sit	0.23	0.60	0.32	0.20	0.00	0.01	0.08	0.00	0.02	0.00	0.05	0.06
sitToStand	0.00	0.00	0.00	0.01	0.00	0.00	0.00	0.00	0.00	0.00	0.00	0.00

Table S14: Transforming gyroscope or accelerometer data to a standard frame of reference (having the phone upside down and with the phone's screen facing the leg). For a given axis, the data is either left unchanged (indicated by 1) or multiplied by -1 (indicated by -1).

Screen Facing Leg?	Upside Down?	x	y	z
no	no	1	-1	-1
no	yes	-1	1	-1
yes	no	-1	-1	1
yes	yes	1	1	1

Table S15: Front pocket gyroscope: Distribution of predicted activity label during test segment where the phone was upside down with its screen not facing the leg (using L_2 distance as the distance metric). *Subtables (a)-(d) correspond to Participants 1-4, respectively. In each subtable, the column headings show the true activity label and the row headings show the predicted activity label. There are two subcolumns under each column heading, the first for classifications based on tri-axial data and the second for classifications based on magnitude data. Each subcolumn gives the distribution of predicted activity labels, when the true activity label is that given by the column heading. Thus, each subcolumn sums to 1. The shaded diagonal highlights the proportion of predicted activity labels that match the true activity label. The average of the diagonal elements for each data type (tri-axial vs. magnitude) is 0.28 vs. 0.71 for Participant 1, 0.39 vs. 0.52 for Participant 2, 0.35 vs. 0.54 for Participant 3, and 0.30 vs. 0.57 for Participant 4.*

(a) Participant 1

	stand		walk		stairUp		stairDown	
stand	0.35	0.15	0.00	0.00	0.00	0.00	0.00	0.00
walk	0.00	0.00	0.01	0.89	0.00	0.00	0.50	0.03
stairUp	0.00	0.00	0.56	0.09	0.77	1.00	0.45	0.15
stairDown	0.00	0.10	0.41	0.01	0.00	0.00	0.00	0.81
standToSit	0.00	0.30	0.01	0.00	0.18	0.00	0.06	0.00
sit	0.65	0.45	0.00	0.00	0.04	0.00	0.00	0.00
sitToStand	0.00	0.00	0.01	0.00	0.02	0.00	0.00	0.00

(b) Participant 2

	stand		walk		stairUp		stairDown	
stand	0.00	0.00	0.00	0.00	0.00	0.00	0.00	0.00
walk	0.00	0.00	0.82	0.71	0.22	0.04	0.80	0.04
stairUp	0.00	0.50	0.01	0.23	0.55	0.83	0.00	0.42
stairDown	0.40	0.00	0.16	0.06	0.22	0.12	0.19	0.54
standToSit	0.00	0.05	0.01	0.00	0.00	0.00	0.00	0.00
sit	0.35	0.10	0.00	0.00	0.00	0.00	0.00	0.00
sitToStand	0.25	0.35	0.00	0.00	0.00	0.00	0.01	0.00

(c) Participant 3

	stand		walk		stairUp		stairDown	
stand	0.16	0.21	0.00	0.00	0.00	0.00	0.00	0.00
walk	0.00	0.00	0.10	0.72	0.19	0.25	0.05	0.10
stairUp	0.00	0.01	0.09	0.07	0.27	0.60	0.05	0.28
stairDown	0.01	0.00	0.81	0.21	0.53	0.15	0.85	0.62
standToSit	0.00	0.10	0.00	0.00	0.00	0.00	0.00	0.00
sit	0.83	0.69	0.00	0.00	0.00	0.00	0.05	0.00
sitToStand	0.00	0.00	0.00	0.00	0.00	0.00	0.00	0.00

(d) Participant 4

	stand		walk		stairUp		stairDown	
stand	0.25	0.25	0.00	0.00	0.00	0.00	0.00	0.00
walk	0.00	0.03	0.00	0.90	0.64	0.05	0.11	0.57
stairUp	0.00	0.00	0.96	0.10	0.36	0.95	0.31	0.26
stairDown	0.10	0.03	0.04	0.01	0.00	0.00	0.59	0.17
standToSit	0.00	0.00	0.00	0.00	0.00	0.00	0.00	0.00
sit	0.65	0.65	0.00	0.00	0.00	0.00	0.00	0.00
sitToStand	0.00	0.05	0.00	0.00	0.00	0.00	0.00	0.00

Table S16: Front pocket accelerometer: Distribution of predicted activity label during test segment where the phone was upside down with its screen not facing the leg (using L_2 distance as the distance metric). *See Table S15 for details. The average of the diagonal elements for each data type (tri-axial vs. magnitude) is 0.45 vs. 0.71 for Participant 1, 0.25 vs. 0.50 for Participant 2, 0.46 vs. 0.75 for Participant 3, and 0.15 vs. 0.73 for Participant 4.*

(a) Participant 1

	stand		walk		stairUp		stairDown	
stand	1.00	0.60	0.08	0.00	0.59	0.00	0.43	0.00
walk	0.00	0.00	0.70	0.50	0.41	0.07	0.48	0.10
stairUp	0.00	0.00	0.00	0.36	0.00	0.91	0.00	0.06
stairDown	0.00	0.00	0.22	0.11	0.00	0.00	0.09	0.84
standToSit	0.00	0.10	0.00	0.02	0.00	0.02	0.00	0.00
sit	0.00	0.30	0.00	0.00	0.00	0.00	0.00	0.00
sitToStand	0.00	0.00	0.00	0.00	0.00	0.00	0.00	0.00

(b) Participant 2

	stand		walk		stairUp		stairDown	
stand	0.00	0.00	0.00	0.00	0.00	0.00	0.00	0.00
walk	0.00	0.60	0.00	0.96	0.00	0.35	0.00	0.40
stairUp	1.00	0.00	1.00	0.02	1.00	0.42	1.00	0.00
stairDown	0.00	0.00	0.00	0.01	0.00	0.13	0.00	0.60
standToSit	0.00	0.00	0.00	0.00	0.00	0.00	0.00	0.00
sit	0.00	0.40	0.00	0.00	0.00	0.00	0.00	0.00
sitToStand	0.00	0.00	0.00	0.00	0.00	0.10	0.00	0.00

(c) Participant 3

	stand		walk		stairUp		stairDown	
stand	1.00	0.91	0.12	0.00	0.51	0.00	0.05	0.00
walk	0.00	0.02	0.84	0.62	0.43	0.07	0.91	0.45
stairUp	0.00	0.00	0.01	0.17	0.00	0.93	0.00	0.03
stairDown	0.00	0.00	0.00	0.21	0.00	0.00	0.00	0.52
standToSit	0.00	0.02	0.03	0.00	0.07	0.00	0.04	0.00
sit	0.00	0.00	0.00	0.00	0.00	0.00	0.00	0.00
sitToStand	0.00	0.05	0.00	0.00	0.00	0.00	0.00	0.00

(d) Participant 4

	stand		walk		stairUp		stairDown	
stand	0.00	0.89	0.00	0.00	0.00	0.00	0.00	0.00
walk	0.12	0.00	0.58	0.75	0.08	0.07	0.61	0.47
stairUp	0.00	0.10	0.00	0.21	0.00	0.89	0.00	0.15
stairDown	0.00	0.01	0.03	0.03	0.00	0.05	0.00	0.38
standToSit	0.00	0.00	0.00	0.00	0.00	0.00	0.00	0.00
sit	0.88	0.00	0.39	0.00	0.92	0.00	0.39	0.00
sitToStand	0.00	0.00	0.00	0.00	0.00	0.00	0.00	0.00

Table S17: Front pocket gyroscope: Distribution of predicted activity label during test segment where the phone was right-side-up with its screen facing the leg (using L_2 distance as the distance metric). *See Table S15 for details. The average of the diagonal elements for each data type (triaxial vs. magnitude) is 0.28 vs. 0.79 for Participant 1, 0.36 vs. 0.47 for Participant 2, 0.38 vs. 0.59 for Participant 3, and 0.54 vs. 0.63 for Participant 4.*

(a) Participant 1

	stand		walk		stairUp		stairDown	
stand	0.10	0.50	0.00	0.00	0.00	0.00	0.00	0.00
walk	0.00	0.00	0.03	0.89	0.00	0.00	0.25	0.01
stairUp	0.30	0.00	0.55	0.11	0.97	1.00	0.73	0.22
stairDown	0.00	0.20	0.42	0.00	0.00	0.00	0.02	0.76
standToSit	0.00	0.00	0.00	0.00	0.03	0.00	0.00	0.00
sit	0.60	0.30	0.00	0.00	0.00	0.00	0.00	0.00
sitToStand	0.00	0.00	0.00	0.00	0.00	0.00	0.00	0.00

(b) Participant 2

	stand		walk		stairUp		stairDown	
stand	0.00	0.10	0.00	0.00	0.00	0.00	0.00	0.00
walk	0.00	0.00	0.76	0.75	0.11	0.24	0.88	0.18
stairUp	0.00	0.00	0.02	0.18	0.57	0.41	0.00	0.21
stairDown	0.00	0.00	0.20	0.07	0.32	0.34	0.12	0.61
standToSit	0.00	0.33	0.01	0.00	0.00	0.00	0.00	0.00
sit	0.57	0.52	0.00	0.00	0.00	0.00	0.00	0.00
sitToStand	0.43	0.05	0.01	0.00	0.00	0.00	0.00	0.00

(c) Participant 3

	stand		walk		stairUp		stairDown	
stand	0.00	0.44	0.00	0.00	0.00	0.00	0.00	0.00
walk	0.00	0.00	0.05	0.80	0.43	0.37	0.02	0.35
stairUp	0.00	0.01	0.33	0.06	0.56	0.58	0.05	0.13
stairDown	0.05	0.00	0.63	0.14	0.01	0.04	0.93	0.52
standToSit	0.00	0.13	0.00	0.00	0.00	0.00	0.00	0.00
sit	0.95	0.42	0.00	0.00	0.00	0.00	0.00	0.00
sitToStand	0.00	0.00	0.00	0.00	0.00	0.00	0.00	0.00

(d) Participant 4

	stand		walk		stairUp		stairDown	
stand	0.51	0.62	0.00	0.00	0.00	0.00	0.00	0.00
walk	0.00	0.00	0.00	0.87	0.19	0.14	0.00	0.24
stairUp	0.00	0.00	0.96	0.12	0.80	0.86	0.14	0.40
stairDown	0.00	0.08	0.04	0.01	0.01	0.00	0.86	0.18
standToSit	0.00	0.00	0.00	0.00	0.00	0.00	0.00	0.12
sit	0.49	0.30	0.00	0.00	0.00	0.00	0.00	0.00
sitToStand	0.00	0.00	0.00	0.00	0.00	0.00	0.00	0.06

Table S18: Front pocket accelerometer: Distribution of predicted activity label during test segment where the phone was right-side-up with its screen facing the leg (using L_2 distance as the distance metric). *See Table S15 for details. The average of the diagonal elements for each data type (tri-axial vs. magnitude) is 0.40 vs. 0.74 for Participant 1, 0.25 vs. 0.59 for Participant 2, 0.48 vs. 0.81 for Participant 3, and 0.11 vs. 0.69 for Participant 4.*

(a) Participant 1

	stand		walk		stairUp		stairDown	
stand	1.00	0.65	0.37	0.00	0.87	0.00	0.95	0.00
walk	0.00	0.00	0.59	0.49	0.13	0.00	0.05	0.13
stairUp	0.00	0.00	0.00	0.35	0.00	1.00	0.00	0.05
stairDown	0.00	0.00	0.05	0.10	0.00	0.00	0.00	0.82
standToSit	0.00	0.10	0.00	0.05	0.00	0.00	0.00	0.00
sit	0.00	0.25	0.00	0.00	0.00	0.00	0.00	0.00
sitToStand	0.00	0.00	0.00	0.01	0.00	0.00	0.00	0.00

(b) Participant 2

	stand		walk		stairUp		stairDown	
stand	0.00	0.35	0.00	0.00	0.00	0.00	0.00	0.00
walk	0.00	0.00	0.00	0.92	0.00	0.19	0.00	0.19
stairUp	1.00	0.10	1.00	0.04	1.00	0.35	1.00	0.06
stairDown	0.00	0.00	0.00	0.02	0.00	0.40	0.00	0.75
standToSit	0.00	0.00	0.00	0.01	0.00	0.00	0.00	0.00
sit	0.00	0.55	0.00	0.01	0.00	0.00	0.00	0.00
sitToStand	0.00	0.00	0.00	0.00	0.00	0.07	0.00	0.00

(c) Participant 3

	stand		walk		stairUp		stairDown	
stand	0.98	0.93	0.00	0.00	0.26	0.00	0.02	0.00
walk	0.02	0.00	0.36	0.64	0.06	0.00	0.83	0.07
stairUp	0.00	0.02	0.52	0.20	0.45	1.00	0.00	0.24
stairDown	0.00	0.00	0.10	0.16	0.00	0.00	0.15	0.68
standToSit	0.00	0.05	0.01	0.00	0.23	0.00	0.00	0.00
sit	0.00	0.00	0.00	0.00	0.00	0.00	0.00	0.00
sitToStand	0.00	0.00	0.00	0.00	0.00	0.00	0.00	0.00

(d) Participant 4

	stand		walk		stairUp		stairDown	
stand	0.00	0.83	0.00	0.00	0.00	0.00	0.00	0.00
walk	0.00	0.00	0.33	0.76	0.31	0.10	0.47	0.32
stairUp	0.00	0.13	0.00	0.22	0.00	0.68	0.00	0.18
stairDown	0.00	0.00	0.02	0.01	0.00	0.12	0.12	0.50
standToSit	0.00	0.00	0.00	0.00	0.00	0.07	0.00	0.00
sit	1.00	0.00	0.65	0.00	0.69	0.00	0.41	0.00
sitToStand	0.00	0.03	0.00	0.01	0.00	0.04	0.00	0.00

Table S19: Front pocket gyroscope: Distribution of predicted activity label during test segment where the phone was right-side-up with its screen not facing the leg (using L_2 distance as the distance metric). *See Table S15 for details. The average of the diagonal elements for each data type (tri-axial vs. magnitude) is 0.84 vs. 0.84 for Participant 1, 0.63 vs. 0.43 for Participant 2, 0.68 vs. 0.65 for Participant 3, and 0.63 vs. 0.52 for Participant 4.*

(a) Participant 1

	stand		walk		stairUp		stairDown	
stand	0.65	0.70	0.00	0.00	0.00	0.00	0.00	0.00
walk	0.00	0.00	0.74	0.87	0.00	0.00	0.03	0.11
stairUp	0.00	0.00	0.09	0.11	1.00	1.00	0.00	0.11
stairDown	0.00	0.00	0.16	0.02	0.00	0.00	0.97	0.78
standToSit	0.00	0.00	0.01	0.00	0.00	0.00	0.00	0.00
sit	0.35	0.30	0.00	0.00	0.00	0.00	0.00	0.00
sitToStand	0.00	0.00	0.00	0.00	0.00	0.00	0.00	0.00

(b) Participant 2

	stand		walk		stairUp		stairDown	
stand	0.00	0.00	0.00	0.00	0.00	0.00	0.00	0.00
walk	0.00	0.00	0.95	0.77	0.10	0.11	0.27	0.43
stairUp	0.00	0.80	0.03	0.20	0.85	0.68	0.00	0.30
stairDown	0.60	0.10	0.02	0.03	0.05	0.21	0.71	0.27
standToSit	0.00	0.00	0.00	0.00	0.00	0.00	0.00	0.00
sit	0.40	0.10	0.00	0.00	0.00	0.00	0.00	0.00
sitToStand	0.00	0.00	0.00	0.00	0.00	0.00	0.02	0.00

(c) Participant 3

	stand		walk		stairUp		stairDown	
stand	0.07	0.53	0.00	0.00	0.00	0.00	0.00	0.00
walk	0.00	0.00	0.80	0.92	0.00	0.18	0.13	0.34
stairUp	0.03	0.11	0.18	0.05	1.00	0.82	0.00	0.32
stairDown	0.05	0.00	0.02	0.03	0.00	0.00	0.87	0.33
standToSit	0.00	0.00	0.00	0.00	0.00	0.00	0.00	0.00
sit	0.85	0.36	0.00	0.00	0.00	0.00	0.00	0.00
sitToStand	0.00	0.00	0.00	0.00	0.00	0.00	0.00	0.00

(d) Participant 4

	stand		walk		stairUp		stairDown	
stand	0.12	0.30	0.00	0.00	0.00	0.00	0.00	0.00
walk	0.00	0.00	0.96	0.88	0.19	0.20	0.33	0.60
stairUp	0.00	0.00	0.03	0.12	0.81	0.80	0.04	0.32
stairDown	0.08	0.00	0.01	0.00	0.00	0.00	0.63	0.08
standToSit	0.00	0.00	0.00	0.00	0.00	0.00	0.00	0.00
sit	0.80	0.65	0.00	0.00	0.00	0.00	0.00	0.00
sitToStand	0.00	0.04	0.00	0.00	0.00	0.00	0.00	0.00

Table S20: Front pocket accelerometer: Distribution of predicted activity label during test segment where the phone was right-side-up with its screen not facing the leg (using L_2 distance as the distance metric). *See Table S15 for details. The average of the diagonal elements for each data type (tri-axial vs. magnitude) is 0.71 vs. 0.72 for Participant 1, 0.16 vs. 0.52 for Participant 2, 0.65 vs. 0.83 for Participant 3, and 0.46 vs. 0.74 for Participant 4.*

(a) Participant 1

	stand		walk		stairUp		stairDown	
stand	1.00	0.45	0.02	0.00	0.00	0.00	0.25	0.00
walk	0.00	0.00	0.59	0.48	0.00	0.00	0.12	0.00
stairUp	0.00	0.00	0.23	0.41	0.93	1.00	0.34	0.06
stairDown	0.00	0.00	0.15	0.08	0.00	0.00	0.29	0.94
standToSit	0.00	0.00	0.01	0.02	0.07	0.00	0.00	0.00
sit	0.00	0.55	0.00	0.00	0.00	0.00	0.00	0.00
sitToStand	0.00	0.00	0.00	0.01	0.00	0.00	0.00	0.00

(b) Participant 2

	stand		walk		stairUp		stairDown	
stand	0.00	0.00	0.00	0.00	0.00	0.00	0.00	0.00
walk	0.00	0.90	0.07	0.96	0.04	0.65	0.00	0.18
stairUp	0.00	0.00	0.00	0.01	0.00	0.28	0.00	0.00
stairDown	0.50	0.10	0.01	0.01	0.42	0.07	0.57	0.82
standToSit	0.00	0.00	0.09	0.01	0.21	0.00	0.11	0.00
sit	0.50	0.00	0.83	0.00	0.33	0.00	0.32	0.00
sitToStand	0.00	0.00	0.00	0.01	0.00	0.00	0.00	0.00

(c) Participant 3

	stand		walk		stairUp		stairDown	
stand	0.87	0.90	0.00	0.00	0.07	0.00	0.00	0.00
walk	0.01	0.00	0.84	0.67	0.06	0.00	0.58	0.18
stairUp	0.01	0.10	0.11	0.17	0.80	1.00	0.32	0.07
stairDown	0.00	0.00	0.05	0.15	0.00	0.00	0.10	0.75
standToSit	0.11	0.00	0.00	0.00	0.07	0.00	0.00	0.00
sit	0.00	0.00	0.00	0.00	0.00	0.00	0.00	0.00
sitToStand	0.00	0.00	0.00	0.00	0.00	0.00	0.00	0.00

(d) Participant 4

	stand		walk		stairUp		stairDown	
stand	0.00	0.95	0.00	0.00	0.00	0.00	0.00	0.00
walk	0.00	0.00	0.88	0.77	0.20	0.38	0.35	0.17
stairUp	0.00	0.00	0.07	0.21	0.59	0.59	0.26	0.17
stairDown	0.00	0.05	0.04	0.02	0.00	0.04	0.35	0.65
standToSit	0.00	0.00	0.00	0.00	0.00	0.00	0.00	0.00
sit	1.00	0.00	0.01	0.00	0.21	0.00	0.03	0.00
sitToStand	0.00	0.00	0.00	0.00	0.00	0.00	0.00	0.00

Table S21: Front pocket gyroscope: Distribution of predicted activity label during test segment where the phone was upside down with its screen not facing the leg (using correlation as the distance metric). *See Table S15 for details. The average of the diagonal elements for each data type (tri-axial vs. magnitude) is 0.03 vs. 0.74 for Participant 1, 0.31 vs. 0.48 for Participant 2, 0.15 vs. 0.32 for Participant 3, and 0.14 vs. 0.54 for Participant 4.*

(a) Participant 1

	stand		walk		stairUp		stairDown	
stand	0.10	0.55	0.08	0.02	0.26	0.00	0.00	0.00
walk	0.15	0.00	0.01	0.65	0.02	0.00	0.01	0.10
stairUp	0.00	0.25	0.01	0.08	0.00	1.00	0.00	0.03
stairDown	0.00	0.20	0.00	0.13	0.00	0.00	0.00	0.76
standToSit	0.00	0.00	0.04	0.02	0.16	0.00	0.00	0.00
sit	0.75	0.00	0.86	0.10	0.56	0.00	0.99	0.10
sitToStand	0.00	0.00	0.00	0.00	0.00	0.00	0.00	0.00

(b) Participant 2

	stand		walk		stairUp		stairDown	
stand	0.05	0.50	0.00	0.01	0.02	0.04	0.01	0.08
walk	0.00	0.00	0.54	0.57	0.07	0.04	0.04	0.09
stairUp	0.65	0.00	0.01	0.04	0.54	0.41	0.00	0.13
stairDown	0.30	0.25	0.08	0.19	0.26	0.12	0.12	0.43
standToSit	0.00	0.00	0.01	0.00	0.00	0.00	0.00	0.00
sit	0.00	0.25	0.37	0.18	0.08	0.38	0.82	0.27
sitToStand	0.00	0.00	0.00	0.00	0.04	0.00	0.00	0.00

(c) Participant 3

	stand		walk		stairUp		stairDown	
stand	0.21	0.16	0.06	0.10	0.07	0.20	0.36	0.18
walk	0.12	0.00	0.16	0.48	0.43	0.31	0.11	0.06
stairUp	0.12	0.21	0.04	0.06	0.17	0.26	0.03	0.10
stairDown	0.04	0.08	0.52	0.14	0.19	0.14	0.05	0.38
standToSit	0.00	0.00	0.00	0.00	0.07	0.00	0.00	0.00
sit	0.46	0.54	0.21	0.19	0.06	0.09	0.32	0.28
sitToStand	0.07	0.00	0.00	0.03	0.00	0.00	0.14	0.00

(d) Participant 4

	stand		walk		stairUp		stairDown	
stand	0.35	0.28	0.13	0.03	0.41	0.21	0.17	0.13
walk	0.14	0.00	0.00	0.84	0.00	0.09	0.07	0.26
stairUp	0.05	0.05	0.68	0.07	0.11	0.70	0.22	0.09
stairDown	0.01	0.06	0.00	0.02	0.00	0.00	0.09	0.34
standToSit	0.07	0.08	0.07	0.00	0.31	0.00	0.09	0.00
sit	0.38	0.51	0.10	0.04	0.17	0.00	0.36	0.17
sitToStand	0.00	0.02	0.00	0.00	0.00	0.00	0.00	0.00

Table S22: Front pocket accelerometer: Distribution of predicted activity label during test segment where the phone was upside down with its screen not facing the leg (using correlation as the distance metric). *See Table S15 for details. The average of the diagonal elements for each data type (triaxial vs. magnitude) is 0.07 vs. 0.56 for Participant 1, 0.18 vs. 0.51 for Participant 2, 0.19 vs. 0.47 for Participant 3, and 0.16 vs. 0.51 for Participant 4.*

(a) Participant 1

	stand		walk		stairUp		stairDown	
stand	0.00	0.00	0.04	0.01	0.09	0.00	0.26	0.00
walk	0.00	0.15	0.07	0.75	0.21	0.23	0.10	0.15
stairUp	0.60	0.00	0.30	0.04	0.10	0.71	0.15	0.09
stairDown	0.00	0.00	0.32	0.03	0.51	0.00	0.13	0.77
standToSit	0.00	0.00	0.00	0.00	0.00	0.00	0.00	0.00
sit	0.35	0.85	0.27	0.16	0.10	0.06	0.37	0.00
sitToStand	0.05	0.00	0.00	0.00	0.00	0.00	0.00	0.00

(b) Participant 2

	stand		walk		stairUp		stairDown	
stand	0.00	0.10	0.02	0.00	0.20	0.13	0.19	0.00
walk	0.00	0.25	0.11	0.96	0.00	0.38	0.27	0.27
stairUp	0.30	0.00	0.22	0.02	0.27	0.35	0.02	0.00
stairDown	0.20	0.10	0.24	0.01	0.21	0.06	0.36	0.61
standToSit	0.00	0.40	0.00	0.00	0.00	0.00	0.00	0.00
sit	0.50	0.15	0.42	0.01	0.32	0.04	0.17	0.12
sitToStand	0.00	0.00	0.00	0.00	0.00	0.04	0.00	0.00

(c) Participant 3

	stand		walk		stairUp		stairDown	
stand	0.36	0.08	0.28	0.00	0.29	0.00	0.00	0.00
walk	0.14	0.43	0.00	0.73	0.00	0.22	0.00	0.31
stairUp	0.00	0.00	0.40	0.05	0.05	0.56	0.22	0.05
stairDown	0.12	0.25	0.19	0.16	0.36	0.10	0.35	0.49
standToSit	0.00	0.00	0.00	0.00	0.00	0.00	0.00	0.00
sit	0.38	0.23	0.13	0.07	0.30	0.12	0.43	0.15
sitToStand	0.00	0.00	0.00	0.00	0.00	0.00	0.00	0.00

(d) Participant 4

	stand		walk		stairUp		stairDown	
stand	0.13	0.16	0.13	0.03	0.22	0.00	0.24	0.14
walk	0.33	0.15	0.10	0.79	0.03	0.11	0.02	0.28
stairUp	0.08	0.14	0.04	0.14	0.21	0.67	0.00	0.02
stairDown	0.00	0.28	0.05	0.04	0.08	0.17	0.20	0.42
standToSit	0.00	0.00	0.01	0.00	0.00	0.00	0.00	0.00
sit	0.37	0.28	0.68	0.01	0.46	0.05	0.54	0.14
sitToStand	0.10	0.00	0.01	0.00	0.00	0.00	0.00	0.00

Table S23: Front pocket gyroscope: Distribution of predicted activity label during test segment where the phone was right-side-up with its screen facing the leg (using correlation as the distance metric). *See Table S15 for details. The average of the diagonal elements for each data type (triaxial vs. magnitude) is 0.01 vs. 0.62 for Participant 1, 0.22 vs. 0.30 for Participant 2, 0.11 vs. 0.53 for Participant 3, and 0.15 vs. 0.54 for Participant 4.*

(a) Participant 1

	stand		walk		stairUp		stairDown	
stand	0.00	0.00	0.14	0.02	0.12	0.00	0.01	0.05
walk	0.00	0.00	0.00	0.69	0.00	0.00	0.00	0.07
stairUp	0.35	0.00	0.01	0.11	0.02	1.00	0.00	0.08
stairDown	0.00	0.00	0.01	0.08	0.00	0.00	0.00	0.78
standToSit	0.00	0.00	0.08	0.01	0.07	0.00	0.14	0.00
sit	0.65	0.65	0.75	0.08	0.79	0.00	0.85	0.01
sitToStand	0.00	0.35	0.00	0.00	0.00	0.00	0.00	0.00

(b) Participant 2

	stand		walk		stairUp		stairDown	
stand	0.00	0.00	0.01	0.01	0.19	0.02	0.00	0.13
walk	0.00	0.00	0.56	0.57	0.14	0.06	0.06	0.06
stairUp	0.57	0.05	0.04	0.09	0.28	0.06	0.00	0.00
stairDown	0.00	0.00	0.07	0.20	0.23	0.33	0.05	0.57
standToSit	0.00	0.00	0.00	0.00	0.00	0.00	0.00	0.00
sit	0.43	0.95	0.32	0.12	0.16	0.52	0.89	0.23
sitToStand	0.00	0.00	0.00	0.00	0.00	0.00	0.00	0.00

(c) Participant 3

	stand		walk		stairUp		stairDown	
stand	0.05	0.42	0.04	0.04	0.04	0.12	0.14	0.00
walk	0.01	0.03	0.20	0.64	0.81	0.05	0.19	0.31
stairUp	0.37	0.12	0.16	0.04	0.13	0.71	0.06	0.05
stairDown	0.19	0.00	0.59	0.06	0.00	0.06	0.08	0.36
standToSit	0.00	0.00	0.01	0.00	0.02	0.00	0.00	0.00
sit	0.38	0.43	0.01	0.22	0.00	0.05	0.44	0.28
sitToStand	0.00	0.00	0.00	0.01	0.00	0.00	0.10	0.00

(d) Participant 4

	stand		walk		stairUp		stairDown	
stand	0.21	0.39	0.13	0.00	0.29	0.03	0.51	0.23
walk	0.13	0.03	0.00	0.88	0.00	0.12	0.00	0.04
stairUp	0.00	0.02	0.78	0.08	0.30	0.70	0.03	0.02
stairDown	0.00	0.00	0.00	0.03	0.00	0.06	0.08	0.17
standToSit	0.23	0.00	0.03	0.00	0.20	0.00	0.00	0.00
sit	0.43	0.56	0.06	0.01	0.20	0.10	0.38	0.53
sitToStand	0.00	0.00	0.00	0.00	0.00	0.00	0.00	0.00

Table S24: Front pocket acclerometer: Distribution of predicted activity label during test segment where the phone was right-side-up with its screen facing the leg (using correlation as the distance metric). *See Table S15 for details. The average of the diagonal elements for each data type (triaxial vs. magnitude) is 0.08 vs. 0.62 for Participant 1, 0.27 vs. 0.56 for Participant 2, 0.28 vs. 0.52 for Participant 3, and 0.09 vs. 0.54 for Participant 4.*

(a) Participant 1

	stand		walk		stairUp		stairDown	
stand	0.00	0.25	0.08	0.08	0.40	0.00	0.11	0.00
walk	0.00	0.00	0.06	0.71	0.21	0.20	0.11	0.09
stairUp	0.30	0.00	0.45	0.05	0.11	0.73	0.47	0.02
stairDown	0.15	0.50	0.20	0.06	0.01	0.01	0.15	0.81
standToSit	0.00	0.00	0.00	0.00	0.00	0.00	0.00	0.00
sit	0.55	0.25	0.21	0.10	0.27	0.07	0.17	0.08
sitToStand	0.00	0.00	0.00	0.00	0.00	0.00	0.00	0.00

(b) Participant 2

	stand		walk		stairUp		stairDown	
stand	0.40	0.00	0.03	0.00	0.19	0.00	0.33	0.00
walk	0.25	0.30	0.26	0.94	0.03	0.13	0.07	0.15
stairUp	0.00	0.00	0.13	0.02	0.29	0.49	0.22	0.00
stairDown	0.00	0.00	0.19	0.01	0.23	0.31	0.15	0.79
standToSit	0.10	0.00	0.00	0.01	0.00	0.00	0.00	0.00
sit	0.25	0.30	0.38	0.00	0.26	0.07	0.22	0.06
sitToStand	0.00	0.40	0.00	0.00	0.00	0.00	0.00	0.00

(c) Participant 3

	stand		walk		stairUp		stairDown	
stand	0.40	0.22	0.20	0.00	0.46	0.00	0.17	0.00
walk	0.09	0.29	0.04	0.66	0.00	0.02	0.17	0.23
stairUp	0.05	0.08	0.32	0.04	0.29	0.83	0.04	0.08
stairDown	0.15	0.19	0.40	0.16	0.17	0.15	0.40	0.37
standToSit	0.00	0.00	0.00	0.00	0.00	0.00	0.00	0.00
sit	0.32	0.23	0.04	0.13	0.07	0.00	0.23	0.33
sitToStand	0.00	0.00	0.00	0.00	0.00	0.00	0.00	0.00

(d) Participant 4

	stand		walk		stairUp		stairDown	
stand	0.00	0.20	0.15	0.05	0.00	0.01	0.20	0.11
walk	0.17	0.37	0.11	0.81	0.00	0.04	0.09	0.23
stairUp	0.15	0.27	0.12	0.10	0.19	0.59	0.20	0.11
stairDown	0.00	0.12	0.07	0.02	0.02	0.18	0.06	0.55
standToSit	0.12	0.00	0.00	0.00	0.00	0.00	0.00	0.00
sit	0.47	0.05	0.53	0.01	0.79	0.18	0.45	0.00
sitToStand	0.10	0.00	0.02	0.00	0.00	0.00	0.00	0.00

Table S25: Front pocket gyroscope: Distribution of predicted activity label during test segment where the phone was right-side-up with its screen not facing the leg (using correlation as the distance metric). *See Table S15 for details. The average of the diagonal elements for each data type (tri-axial vs. magnitude) is 0.73 vs. 0.67 for Participant 1, 0.51 vs. 0.45 for Participant 2, 0.65 vs. 0.44 for Participant 3, and 0.62 vs. 0.47 for Participant 4.*

(a) Participant 1

	stand		walk		stairUp		stairDown	
stand	0.25	0.30	0.01	0.01	0.05	0.00	0.00	0.00
walk	0.00	0.15	0.80	0.53	0.04	0.00	0.05	0.05
stairUp	0.00	0.00	0.09	0.08	0.91	1.00	0.00	0.03
stairDown	0.00	0.00	0.08	0.21	0.00	0.00	0.95	0.83
standToSit	0.70	0.40	0.00	0.02	0.00	0.00	0.00	0.00
sit	0.05	0.00	0.00	0.15	0.00	0.00	0.00	0.09
sitToStand	0.00	0.15	0.02	0.00	0.00	0.00	0.00	0.00

(b) Participant 2

	stand		walk		stairUp		stairDown	
stand	0.00	0.50	0.00	0.00	0.00	0.00	0.14	0.03
walk	0.00	0.00	0.68	0.68	0.00	0.00	0.22	0.25
stairUp	0.00	0.00	0.03	0.05	0.85	0.11	0.01	0.00
stairDown	0.50	0.00	0.02	0.11	0.02	0.52	0.52	0.52
standToSit	0.00	0.00	0.00	0.00	0.00	0.00	0.00	0.00
sit	0.50	0.50	0.26	0.16	0.13	0.37	0.12	0.20
sitToStand	0.00	0.00	0.00	0.00	0.00	0.00	0.00	0.00

(c) Participant 3

	stand		walk		stairUp		stairDown	
stand	0.17	0.14	0.00	0.05	0.00	0.00	0.00	0.05
walk	0.09	0.00	0.81	0.76	0.00	0.34	0.14	0.25
stairUp	0.34	0.47	0.18	0.03	0.98	0.66	0.12	0.08
stairDown	0.16	0.00	0.01	0.03	0.00	0.00	0.62	0.22
standToSit	0.00	0.05	0.00	0.00	0.00	0.00	0.00	0.00
sit	0.24	0.34	0.01	0.13	0.02	0.00	0.12	0.41
sitToStand	0.00	0.00	0.00	0.00	0.00	0.00	0.00	0.00

(d) Participant 4

	stand		walk		stairUp		stairDown	
stand	0.20	0.13	0.00	0.00	0.00	0.04	0.00	0.36
walk	0.29	0.21	0.96	0.91	0.12	0.23	0.36	0.20
stairUp	0.17	0.07	0.03	0.07	0.88	0.68	0.00	0.07
stairDown	0.04	0.03	0.01	0.00	0.00	0.04	0.45	0.18
standToSit	0.12	0.00	0.00	0.00	0.00	0.00	0.00	0.00
sit	0.17	0.57	0.00	0.02	0.00	0.00	0.19	0.19
sitToStand	0.00	0.00	0.00	0.00	0.00	0.00	0.00	0.00

Table S26: Front pocket accelerometer: Distribution of predicted activity label during test segment where the phone was right-side-up with its screen not facing the leg (using correlation as the distance metric). *See Table S15 for details. The average of the diagonal elements for each data type (tri-axial vs. magnitude) is 0.46 vs. 0.48 for Participant 1, 0.44 vs. 0.50 for Participant 2, 0.43 vs. 0.63 for Participant 3, and 0.39 vs. 0.48 for Participant 4.*

(a) Participant 1

	stand		walk		stairUp		stairDown	
stand	0.40	0.00	0.04	0.15	0.07	0.00	0.12	0.02
walk	0.00	0.00	0.73	0.62	0.17	0.06	0.07	0.00
stairUp	0.00	0.10	0.03	0.08	0.46	0.44	0.32	0.05
stairDown	0.00	0.00	0.11	0.02	0.20	0.00	0.26	0.88
standToSit	0.00	0.30	0.00	0.00	0.00	0.00	0.00	0.00
sit	0.60	0.60	0.09	0.13	0.10	0.50	0.23	0.05
sitToStand	0.00	0.00	0.00	0.00	0.00	0.00	0.00	0.00

(b) Participant 2

	stand		walk		stairUp		stairDown	
stand	0.50	0.00	0.24	0.00	0.03	0.07	0.06	0.00
walk	0.00	0.80	0.55	0.96	0.15	0.38	0.06	0.20
stairUp	0.00	0.00	0.01	0.01	0.09	0.26	0.23	0.03
stairDown	0.50	0.10	0.11	0.01	0.41	0.12	0.60	0.77
standToSit	0.00	0.00	0.00	0.01	0.00	0.17	0.00	0.00
sit	0.00	0.00	0.09	0.01	0.32	0.00	0.06	0.00
sitToStand	0.00	0.10	0.00	0.00	0.00	0.00	0.00	0.00

(c) Participant 3

	stand		walk		stairUp		stairDown	
stand	0.29	0.51	0.00	0.01	0.04	0.00	0.00	0.00
walk	0.22	0.18	0.83	0.65	0.27	0.00	0.56	0.13
stairUp	0.04	0.00	0.08	0.13	0.46	0.83	0.27	0.09
stairDown	0.02	0.09	0.06	0.10	0.03	0.05	0.13	0.51
standToSit	0.14	0.00	0.00	0.00	0.00	0.00	0.00	0.00
sit	0.29	0.23	0.03	0.11	0.20	0.12	0.05	0.27
sitToStand	0.00	0.00	0.00	0.00	0.00	0.00	0.00	0.00

(d) Participant 4

	stand		walk		stairUp		stairDown	
stand	0.09	0.24	0.00	0.02	0.00	0.00	0.16	0.07
walk	0.19	0.09	0.88	0.79	0.21	0.50	0.30	0.17
stairUp	0.20	0.16	0.02	0.16	0.41	0.25	0.16	0.03
stairDown	0.02	0.15	0.00	0.02	0.03	0.14	0.20	0.62
standToSit	0.03	0.11	0.00	0.00	0.00	0.00	0.00	0.00
sit	0.41	0.24	0.10	0.01	0.36	0.10	0.17	0.11
sitToStand	0.07	0.00	0.00	0.00	0.00	0.00	0.00	0.00
